# Honey Phenolic Compound Profiling and Authenticity Assessment Using HRMS Targeted and Untargeted Metabolomics

**DOI:** 10.3390/molecules26092769

**Published:** 2021-05-08

**Authors:** Georgios A. Koulis, Aristeidis S. Tsagkaris, Reza Aalizadeh, Marilena E. Dasenaki, Eleni I. Panagopoulou, Spyros Drivelos, Michał Halagarda, Constantinos A. Georgiou, Charalampos Proestos, Nikolaos S. Thomaidis

**Affiliations:** 1 Analytical Chemistry Laboratory, Chemistry Department, National and Kapodistrian University of Athens, Panepistimiopolis Zographou, 15771 Athens, Greece; georgekoulis@chem.uoa.gr (G.A.K.); tsagkara@vscht.cz (A.S.T.); raalizadeh@chem.uoa.gr (R.A.); elenapanag@chem.uoa.gr (E.I.P.); ntho@chem.uoa.gr (N.S.T.); 2 Food Chemistry Laboratory, Department of Chemistry, National and Kapodistrian University of Athens, Panepistimiopolis Zographou, 15771 Athens, Greece; harpro@chem.uoa.gr; 3 Department of Food Analysis and Nutrition, Faculty of Food and Biochemical Technology, University of Chemistry and Technology Prague, Technická 5, Prague 6—Dejvice, 16628 Prague, Czech Republic; 4 Chemistry Laboratory, Department of Food Science and Human Nutrition, Agricultural University of Athens, 75 Iera Odos, 11855 Athens, Greece; sdrivelos@aua.gr (S.D.); cag@aua.gr (C.A.G.); 5 Department of Food Product Quality, Cracow University of Economics, ul. Sienkiewicza 5, 30033 Krakow, Poland; michal.halagarda@uek.krakow.pl

**Keywords:** honey, authenticity, phenolic compounds, metabolomics, UPLC–QToF-MS, origin discrimination, geographical origin

## Abstract

Honey consumption is attributed to potentially advantageous effects on human health due to its antioxidant capacity as well as anti-inflammatory and antimicrobial activity, which are mainly related to phenolic compound content. Phenolic compounds are secondary metabolites of plants, and their content in honey is primarily affected by the botanical and geographical origin. In this study, a high-resolution mass spectrometry (HRMS) method was applied to determine the phenolic profile of various honey matrices and investigate authenticity markers. A fruitful sample set was collected, including honey from 10 different botanical sources (*n* = 51) originating from Greece and Poland. Generic liquid–liquid extraction using ethyl acetate as the extractant was used to apply targeted and non-targeted workflows simultaneously. The method was fully validated according to the Eurachem guidelines, and it demonstrated high accuracy, precision, and sensitivity resulting in the detection of 11 target analytes in the samples. Suspect screening identified 16 bioactive compounds in at least one sample, with abscisic acid isomers being the most abundant in arbutus honey. Importantly, 10 markers related to honey geographical origin were revealed through non-targeted screening and the application of advanced chemometric tools. In conclusion, authenticity markers and discrimination patterns were emerged using targeted and non-targeted workflows, indicating the impact of this study on food authenticity and metabolomic fields.

## 1. Introduction

The development of analytical methods for verifying food origin is an emerging field that is rapidly developing [1]. Honey is a foodstuff highly consumed globally as it is connected with several health benefits [2]. Botanical and geographical origin decisively affect the honey market price. For example, there is an ever-increasing demand for honeydew honey in many European countries, such as fir honey, resulting in a higher price [3]. Additionally, honey produced in China or South America usually has a lower price than honey produced in Europe due to several fraud incidents that have been reported during the previous years in these markets. Differences in honey quality and price are also observed among European countries—honey from southern countries is usually more attractive due to the unique characteristics attributed to the Mediterranean climate—and even within the same country, honey quality varies among different geographical regions. To this end, analytical methodologies capable of discriminating the geographical origin of honey are much needed [4]. 

To begin with, melissopalynological analysis is the traditional way to investigate honey authenticity [5]. However, this involves certain drawbacks, with the most important being the substantial deviation of analysis outcome according to the analyst experience, demanding commonly further organoleptic or chemical analysis [6,7]. Additionally, the evaluation of physicochemical characteristics such as water activity, pH, or hydroxymethylfurfural (HMF) is the most straightforward and affordable approach for assessing honey origin [8]. Nevertheless, these methods can be considered complementary as they are usually combined with other analytical techniques to achieve a valuable result [9]. Several studies have been reported demonstrating the applicability of different analytical methods, such as inductively coupled plasma–mass spectrometry (ICP–MS) [10], nuclear magnetic resonance (NMR) [11,12], and fluorescence spectroscopy [13], in the verification of honey geographical origin using various compounds as potential markers. Trace elements [10], stable isotopes [14], volatile organic compounds (VOCs) [15,16] and phenolic compounds [17,18] have been proven to be capable biomarkers for both geographical and botanical discrimination of honey samples. 

Lately, honey phenolic compounds have drawn great attention as they can be used as authenticity markers and, also, their consumption may result in many health beneficial effects [19,20]. Phenolic compounds are secondary metabolites of plants, biosynthesized mainly for protection against stress and oxidative damage and transferred via the nectar to honey [21]. Phenolic content is related to honey botanical sources [2] alongside climatological and geographical aspects [22]. Considering the ambiguous number of polyphenols and their metabolites encountered in honey, a metabolomic approach capable of evaluating its entire polyphenolic profile may be the solution to successfully combine authenticity studies with phenolic compound profiling. Metabolomic approaches present a powerful tool for food fingerprinting when comprehensive food characterization is needed [23]. High-resolution mass spectrometry (HRMS) allows parallel targeted and non-targeted approaches as well as compound elucidation and retrospective data analysis [24]. Furthermore, HRMS features the necessary analytical characteristics such as quantification, sensitivity, and rapid scan speeds, providing a wealth of data. 

Several publications have already presented the determination of honey phenolic compounds using LC–HRMS analytical platforms [25,26,27,28]. However, there is a lack of analytical methods that can be used both for simultaneous targeted quantification of phenolic compounds and untargeted determination of honey metabolic fingerprint for geographical origin discrimination. So far, most targeted approaches reported in the literature could not be used effectively for untargeted metabolomic studies as they include selective clean-up steps, such as solid-phase extraction (SPE), limiting the number of compounds that can be determined. Concurrently, untargeted methodologies mentioned in the literature for floral and geographical origin discrimination of honey samples have not been assessed for the determination of targeted phenolic compounds [22,29,30,31]. In this context, the present study introduces a UPLC–QToF-MS analytical methodology for the targeted identification and quantification of honey phenolic compounds as well as the untargeted metabolomic discrimination of honey sample geographical origin. Target and suspect screening workflows were utilized for the comprehensive phenolic profiling of honey samples, whilst the non-target screening approach, along with the employment of advanced chemometrics tools, were used for the capability of discovering biomarkers related to honey origin and authenticity.

## 2. Results and Discussion 

### 2.1. Method Development and Validation

The extraction efficiency of 3 different extraction media, namely ethyl acetate (EtAc), acetonitrile (ACN), as well as a mixture of EtAc:ACN (1:1, *v*/*v*), was assessed to choose the most effective extractant for phenolic compounds in the honey matrix. A mixture of apigenin, ethyl vanillin, ferulic acid, hydroxytyrosol, luteolin, p-coumaric acid, quercetin, tyrosol, and vanillin was spiked at a concentration of 0.2 mg/kg in the test samples prior to extraction. EtAc and ACN showed great recovery rates, higher than 97% and 88%, respectively, for most of the tested compounds (see Section S1, Table S1 of the ESM). Importantly, EtAc was able to extract phenolic acids efficiently (recoveries of 104% and 100% for ferulic and p-coumaric acid were obtained) in contrast to ACN, in which a lower recovery rate was achieved. This discrepancy can be possibly attributed to the higher distribution coefficients of these compounds to EtAc compared to ACN. The solubility of phenols in different solvents is determined by their stereochemistry (the polar and the nonpolar part inside their molecules) and the intermolecular forces (mainly hydrogen bonds) between them and the solvents [32]. The use of sodium chloride during the extraction may force the phenolic acids into the EtAc phase because of the nonpolar part of their molecules. Therefore, EtAc was chosen as the extractant since satisfactory recovery yields are necessary for phenolic acids, a class of compounds with proven potential to distinguish honey origin [33]. Moreover, the capability of EtAc to extract compounds within a broader range of polarity as well as that it has been previously utilized successfully in other studies for the extraction of phenolic compounds from honey matrix [25,26,34] were taken into consideration for the final choice.

Afterwards, the developed method was validated as it is described in Section 3.6. Great linearity, with correlation coefficients better than 0.98, was achieved for all the analytes. LODs ranged between 0.030 mg kg^−1^ (ferulic acid) and 0.33 mg kg^−1^ (salicylic acid), whilst LOQs fluctuated between 0.091 and 0.99 mg kg^−1^, respectively. Our detection limits were comparable to previous studies [26,35], proving the ability of HRMS to detect analytes at the parts per billion (ppb) level. Regarding precision, a relative standard deviation under repeatability conditions (%RSDr) less than 5% was obtained for most of the analytes, while a relative standard deviation under intermediate precision conditions (%RSD_R_) of less than 10% was found for almost all analytes apart from quercetin. Additionally, the developed method provided satisfactory results in terms of trueness, as most of the analytes demonstrated a recovery value between 72% and 101%. Although honey is a challenging matrix with high sugars amount, there were no significant ionization problems, with a calculated matrix effect (ME%) mostly within the range of −8.5% to 33%. The higher values of ME% were acquired for the early eluting peaks (up to 1.5 min) corresponding to the phenolic acids 4-hydroxybenzoic acid (32%), 3,4-dihydroxybenzoic acid (31%), gallic acid (33%), and caffeic acid (32%). The complete validation data are presented in Table S2 in Section S1 of Electronic Supplementary Material (ESM). Considering the validation results, the developed method was capable of accurately and sensitively detecting the phenolic compounds.

### 2.2. Target Screening

Targeted analysis can provide quantitative data useful for gaining knowledge on the phenolic compound composition of the tested unifloral honey. A comprehensive list, including 21 phenolic compounds for which reference standards were available in our lab, was composed following the criteria described in Section 3.7.1 and is presented in Table S3 of Section S2 of the ESM. Subsequently, this target list was used to screen 10 different unifloral honey matrices (*n* = 51 samples) derived from Greece and Poland. Eleven phenolic compounds were determined—apigenin, cinnamic acid, ferulic acid, luteolin, p-coumaric acid, quercetin, salicylic acid, taxifolin, vanillin, 3,4-dihydroxybenzoic acid, and 4-hydroxybenzoic acid—and their median values are presented in Table 1. Detected concentrations varied broadly, from 0.05 mg kg^−1^ (cinnamic acid in rape honey) to 15 mg kg^−1^ (4-hydroxybenzoic acid in buckwheat honey). 4-Hydroxybenzoic acid, p-coumaric acid and salicylic acid presented the highest concentrations in buckwheat honey, while 3,4-dihydroxybenzoic acid, quercetin, ferulic acid, apigenin, and cinnamic acid showed the highest concentrations in chestnut, fir, acacia, and heather honey, respectively. Regarding p-coumaric acid, it was found to be the most abundant compound in 3 different matrices, namely linden, rape, and thyme, at concentrations fluctuating from 1.1 to 1.5 mg kg^−1^. On the contrary, acacia, arbutus, linden, rape, and thyme honey were found with the least amount of cinnamic acid. Moreover, apigenin, luteolin, and taxifolin were detected at a quantitative level in 4, 3, and 4 honey matrices, respectively. Considering previous studies, Dimitrova et al. [36] determined the phenolic acid profile in various unifloral honeys, with concentrations ranging between 0.3 and 15 mg kg^−1^, which is in line with our results. Among the determined compounds were ferulic, p-coumaric, and salicylic acid in acacia, chestnut, and heather honey. The phenolic content of acacia was similar to our study, while chestnut honey showed a decreased amount of phenolics in our study. Moreover, a higher value of polyphenols was documented by Can et al. [37]. According to Biesaga et al. [38], buckwheat honey exhibits higher content of 4-hydroxybenzoic acid and p-coumaric acid than acacia honey, something which is in agreement with this study. Fluctuations noticed among the different honey matrices are quite reasonable since the phenolic content may be affected by various factors such as climate and geographical origin of the samples. Our results represent a solid and fruitful dataset for a variety of honey matrices, increasing the current knowledge on the occurrence of phenolic compounds in honey.

### 2.3. Suspect Screening

Regarding the suspect screening, 16 out of 60 suspect compounds were identified in at least one of the analyzed honey samples. All the identified compounds showed high mass accuracy, below 2 mDa, and isotopic fit lower than 50 mSigma in all samples. Moreover, only compounds with intensity higher than 1000 counts and peak area higher than 2000 were examined. The difference between the experimental and predicted retention time was below 1.8 min for almost all analytes except for galangin, which belongs to the model’s applicability domain, but its predicted t_R_ cannot be considered reliable [39]. The compounds 2-*trans*,4-*trans*-abscisic acid, 2-*cis*,4-*trans*-abscisic acid, acacetin, chrysin, galangin, homogentisic acid, isorhamnetin, kaempferol, phenyllactic acid, pinocembrin, rosmarinic acid, and sakuranetin were identified by the comparison of experimental MS/MS fragments with MS/MS spectra found in mass spectral libraries such as MassBank of North America or/and in the literature. On the other hand, 3-hydroxybenzoic acid, benzoic acid, dehydrovomifoliol, lumichrome, methyl syringate, and pinobanksin were tentatively identified by comparing experimental MS/MS spectra with MS/MS spectra obtained by the in silico fragmentation tool MetFrag [40]. A detailed presentation of suspect screening results providing information about the identification criteria and the number of samples in which each compound was detected is presented in Table 2.

**Figure 1:**
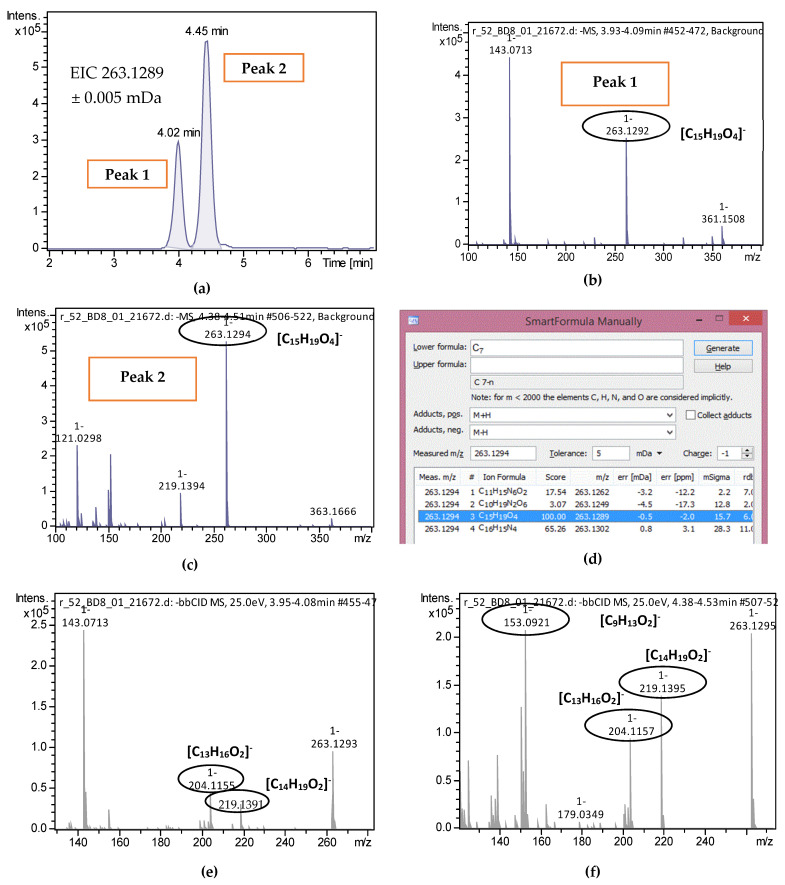

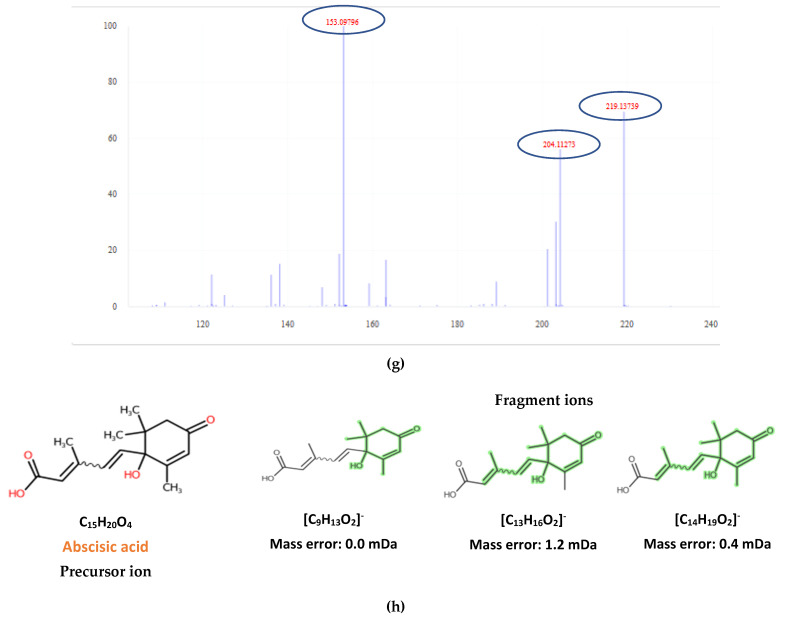
Identification procedure for abscisic acid isomers: (**a**) EIC of *m*/*z* 263.1289 (±5 mDa) in a rape honey; (**b**) Background subtracted MS spectrum from 3.9 to 4.1 min; (**c**) Background subtracted MS spectrum from 4.4 to 4.5 min; (**d**) Molecular formula annotation of *m*/*z* 263.1289; (**e**) Background subtracted MS/MS spectrum from 3.9 to 4.1 min; (**f**) Background subtracted MS/MS spectrum from 4.4 to 4.5 min; (**g**) Fiehn Lab HILIC Library Record FiehnHILIC002566 (abscisic acid); (**h**) Structures of precursor and fragment ions of abscisic acid.

The identification procedure of the suspect compounds was performed as described in Section 3.7.2. The exact workflow for the identification of trans and cis abscisic acid in rape honey is indicatively presented. The extracted ion chromatogram of *m*/*z* 263.1289 corresponding to pseudomolecular ion ([M − H]^−^) of abscisic acid was created by TASQ 1.4, showing two peaks (Figure 1a). The background-subtracted MS spectrum of each peak (Figure 1b,c) was meticulously examined to exclude that the *m*/*z* of interest is not formed as an in-source fragment by any other *m*/*z* exist in the mass spectrum. The most probable molecular formula was suggested by “SmartFormula Manually” to be C_15_H_19_O_4_ in both peaks with a mass error of 0.3 and 0.5 mDa and a mSigma value of 15.1 and 15.7, respectively (Figure 1d). The comparison of experimental MS/MS spectra (Figure 1e,f) to that existing in mass spectral libraries ensued. Specifically, MS/MS spectra of Fiehn Lab HILIC Library, recovered from MassBank of North America, was utilized, as shown in Figure 1g. Two common fragments were revealed in the first peak, while 3 fragments were common in the second peak (Figure 1h). As has already been referred to in the literature, the two abscisic acid isomers can be discriminated by their fragmentation pattern as only 2-*cis*,4-*trans*-abscisic acid gives the fragment with *m*/*z* 153.0922 [41]. Thus, the first peak can be attributed to 2-*trans*,4-*trans*-abscisic acid and the second peak to 2-*cis*,4-*trans*-abscisic acid. Since the *trans*/*trans* and *cis*/*trans* abscisic acid are conformer isomers, predicted t_R_ values estimated by the models could not be distinguishable. The same procedure was followed for all the identified suspect compounds and is presented in Section S3 of ESM (Figures S1–S14).

**Figure 2:**
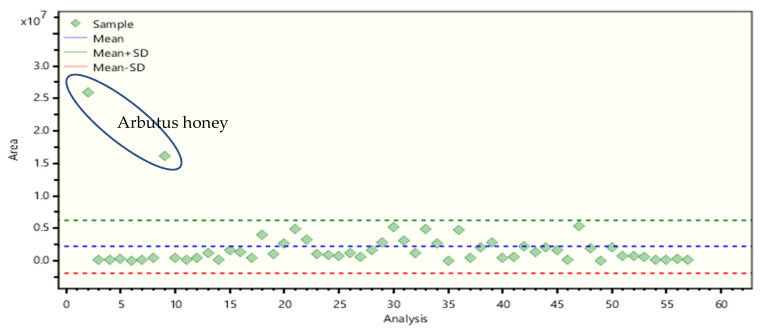
Batch statistics graph showing the mean area and the standard deviation of 2-*cis*,4-*trans*-abscisic acid.

Although abscisic acids are non-phenolic compounds, their presence in honey samples was investigated as they compose a significant part of honey’s bioactive content with many beneficial properties to human health [42]. The isomers of abscisic acid have been reported to encounter in many types of honey, which is also confirmed in this study [27,28,41,43,44,45,46]. Both compounds have been determined in all samples, and in almost all cases, the 2-*cis*,4-*trans*-abscisic acid was more abundant than 2-*trans*,4-*trans*-abscisic acid. The highest abundance of abscisic acid isomers was found in arbutus honey (Figure 2). These findings agree with previous studies in which these compounds have been proposed as floral markers for the *Arbutus unedo* tree [41,47]. Furthermore, a high abundance of abscisic acid isomers was found in heather honey, which is in agreement with that reported in the literature [48].

A detailed presentation showing the number of samples of each botanical origin in which the identified suspect compounds have been detected was presented in Section S3 of ESM (Table S4). Besides abscisic acid isomers, the flavonoids chrysin, galangin, isorhamnetin, pinobanksin, pinocembrin and sakuranetin were also detected in all 51 honey samples. These compounds are among the most important found in honey due to their beneficial properties to human health [20]. Flavonoids are ubiquitous in honey, and thus they have been widely reported in the literature in various types of honey [26,27,46].

Furthermore, the flavonoids acacetin and kaempferol were detected in 42/51 and 45/51 honey samples, respectively. Bobis et al. has proposed acacetin as an authenticity marker of acacia honey [49], while Martos et al. and Tomas-Barberan et al. have suggested kaempferol as a marker for Eucalyptus and Rape honey, respectively [48,50]. In the framework of this study, the authors could not confirm that acacetin is abundant in acacia honey as it was found in low intensity and, in many cases, lower than that in other botanical origins. Moreover, the same is the case for kaempferol, the intensity of which varied among the different botanical sources.

Dehydrovomifoliol, phenyllactic acid, and lumichrome are all non-phenolic compounds that play a significant role in honey’s bioactive content. Dehydrovomifoliol is a norisoprenoid compound that participates in abscisic acid production. This compound has been proposed as an authenticity marker for heather honey [36], especially for Polish honey [51]. These findings come in agreement with this study, where a higher abundance of dehydrovomifoliol has been detected in Polish heather honey in comparison with Greek heather honey, and every other botanical source examined (ESM, Section S3, Figure S15). Phenyllactic acid is an organic acid commonly found in honey, and it was suggested as an authenticity marker for thistle honey [52]. Furthermore, it was referred to exist in high concentration in heather and manuka honey [36,53]. In this study, a higher abundance of this organic acid was revealed in Polish heather honey, as shown in Section S3 of ESM (Figure S16). Finally, lumichrome is the degradation product of vitamin B2 in acidic environment and was first referred to by Tuberoso et al. as a chemical marker of thistle honey [52]. This compound has also been used for discriminating kanuka and manuka honey [54]. High abundance of lumichrome was detected in heather honey, both from Greece and Poland.

A significant group of compounds encountered in honey is phenolic acids and their derivatives. Such compounds are homogentisic acid, rosmarinic acid, and methyl syringate that were detected through suspect screening workflow. Homogentisic acid is valuable for human health because it is attributed to antioxidant and antiradical activities as well as protection against thermal cholesterol degradation [19]. This compound was suggested as a marker of strawberry tree (*Arbutus unedo*) honey in many studies [41,55,56], something that was confirmed in the present study as arbutus honey had the highest abundance (ESM, Section S3, Figure S17). Rosmarinic acid has been proposed as a marker for thyme honey [57]. In this work, rosmarinic acid was detected only in one thyme honey. Methyl syringate is a benzoic acid derivative and has been suggested as a marker for asphodel honey [58] and manuka honey [53]. It was detected in 43 out of 51 honey samples with a higher abundance in Polish rape honey (ESM, Section S3, Figure S18).

### 2.4. Non-Target Screening and Marker Identification

The application of the non-target screening workflow in all 51 Greek and Polish honey samples, as described in Section 3.7.3, resulted in a peak list containing 408 mass features (*m*/*z*_t_R_). Afterwards, PLS-DA was performed to extract the VIP values and identify the most important markers that discriminate Greek from Polish honey samples.

**Figure 3:**
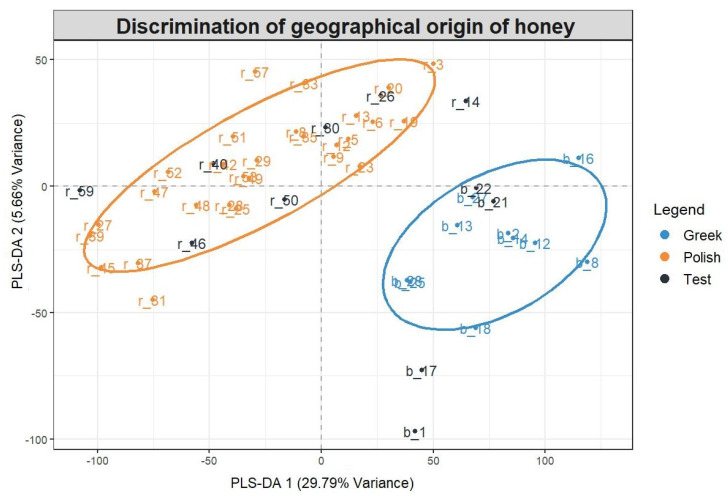
The PLS-DA model for discriminating the geographical origin of honey.

A total of 40 samples were used to study the discrimination between samples (training set). Importantly, the misclassification error rate for the leave-one-out cross validation and training set was 0. A total of 11 independent samples were also used to evaluate the accuracy of the PLS-DA model externally (test set). Figure 3 shows the final PLS-DA model developed along with the test set located within the 95% confidence intervals of each class. In conclusion, the developed PLS-DA model is robust and can be applied to unknown samples to understand their geographical origin with high accuracy.

From the PLS-DA and VIP values, the nine most relevant *m*/*z* features were extracted and identified. These *m*/*z* features were *m*/*z* 191.0566/t_R_ 1.30 min, *m*/*z* 153.0198/t_R_ 1.78 min, *m*/*z* 239.1291/t_R_ 4.21 min, *m*/*z* 313.1803/t_R_ 10.75 min, *m*/*z* 209.0822/t_R_ 3.49 min, *m*/*z* 389.1243/t_R_ 3.71 min, *m*/*z* 253.0512/t_R_ 9.66 min, *m*/*z* 283.0611/t_R_ 10.15 min, *m*/*z* 201.1135/t_R_ 3.94 min, and *m*/*z* 315.0522/t_R_ 7.86 min.

**Figure 4:**
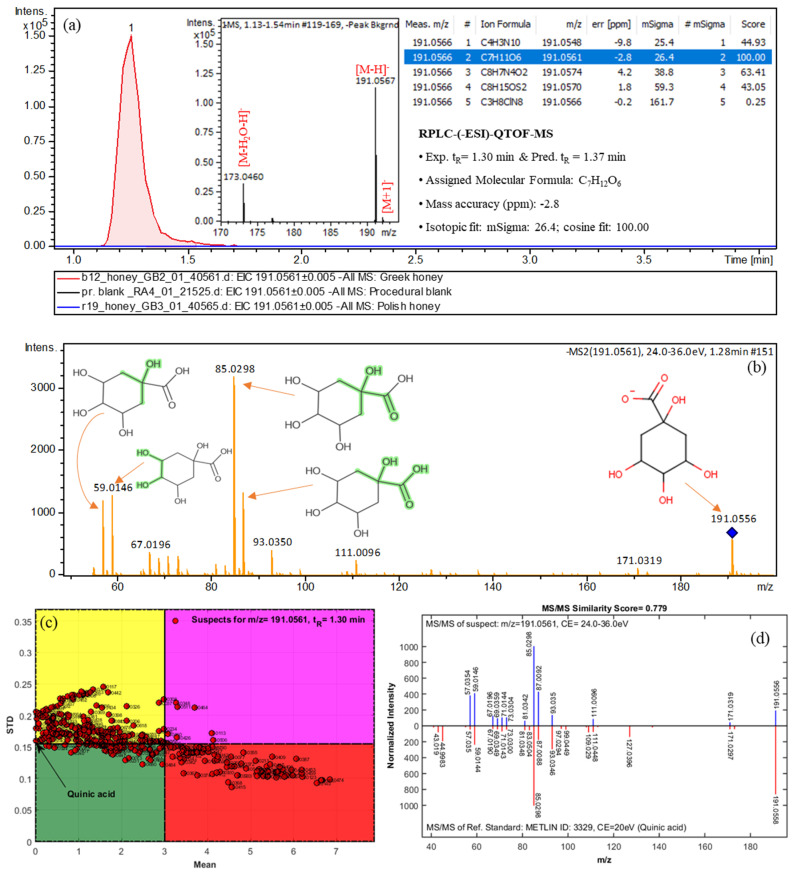
Identification of quinic acid through non-target screening: (**a**) full MS chromatogram and EIC for the given mass (±5 ppm); (**b**) MS/MS spectrum and corresponding fragments; (**c**) MCS plot for evaluating the predicted t_R_ values; (**d**) confirmation step using spectrum from MS library.

To demonstrate the identification methodology, the mass feature with *m*/*z* 191.0566 and t_R_ = 1.30 min was selected (Figure 4). The molecular formula of C_7_H_12_O_6_ was assigned (the mass accuracy and isotopic fit were −2.8 ppm and 26.4 mSigma, respectively, Figure 4a). A total of 702 compounds were retrieved from PubChem with a mass accuracy threshold of 5 ppm. These candidates were then processed by MetFrag [40] and a QSRR-based retention time model [39]. The annotated fragments are given for the most probable candidate (quinic acid) in Figure 4b. The RPLC t_R_ prediction model could help to prioritize these candidates according to their degree of mean value (absolute values of mean of predictive residuals) in the Monte Carlo sampling (MCS) plot (Figure 4c). The spectrum of the reference standard was found in METLIN (METLIN ID: 3329) and the fragments (Figure 4d) at *m*/*z* 57.0354, 59.0146, 67.0196, 69.0359, 71.0144, 73.0304, 81.0342, 85.0298, 87.0092, 93.0350, 111.0096, 171.0319, and 191.0556 fit very well with the prioritized suspect (quinic acid), corresponding to [C_3_H_5_O]^−^, [C_2_H_3_O_2_]^−^, [C_4_H_3_O]^−^, [C_4_H_5_O]^−^, [C_3_H_3_O_2_]^−^, [C_3_H_5_O_2_]^−^, [C_5_H_5_O]^−^, [C_4_H_5_O_2_]^−^, [C_3_H_3_O_3_]^−^, [C_6_H_5_O]^−^, [C_5_H_3_O_3_]^−^, [C_7_H_7_O_5_]^−^, and [C_7_H_11_O_6_]^−^, respectively. Therefore, the identification was confirmed by (i) t_R_ prediction, (ii) MCS plot, (iii) comparison with the available experimental MS/MS fragments in the spectrum library (spectra similarity score of 0.799) and the identification confidence level of 2a (see ESM, Section S8) was assigned.

The list of detected markers in honey according to their geographical origin is provided in Table 3. In detail, for the mass feature detected at *m*/*z* 153.0198, t_R_ = 1.78 min, the molecular formula C_7_H_6_O_4_ was assigned using Bruker “SmartFormula Manually”, according to the criteria of mass accuracy (−2.7 ppm) and isotopic fit (15.9 mSigma). This mass formula was already present in the target list, and it is identified as gentisic acid, which is more abundant in Greek honey samples than in Polish ones. Gentisic acid is the salicylic acid degradation product and has shown anti-inflammatory and antioxidant properties [59].

For the mass feature with *m*/*z* 239.1291, t_R_ = 4.21 min, which is more abundant in Greek honey than Polish honey, the molecular formula of C_13_H_20_O_4_ was assigned with the mass accuracy and isotopic fit of −0.9 ppm and 35.6 mSigma, respectively. A total of 3315 candidates were retrieved from PubChem and processed using MetFrag. This list was furtherly filtered with the QSRR-based retention time model, keeping the candidates with MetFrag score above 0.5 and retention time error below 1.8 min. Overall, 50 candidates remained. Therefore, only one molecular formula can be attributed to this mass feature, and so this marker is identified tentatively at the level of identification 4.

For the mass feature detected at *m*/*z* 313.1803, t_R_ = 10.75 min, the molecular formula of C_20_H_26_O_3_ was assigned with the mass accuracy and isotopic fit of −2.1 ppm and 20.3 mSigma, respectively. A total of 2232 candidates were found in PubChem based on the assigned molecular formula, and further information (such as intense and clean MS/MS fragments) was needed to proceed to higher identification confidence. Therefore, this mass is tentatively identified at the level of identification confidence 4.

For the mass feature with *m*/*z* 209.0822 and t_R_ = 3.49 min, the molecular formula of C_11_H_14_O_4_ was assigned with the mass accuracy and isotopic fit of −1.5 ppm and 26.7 mSigma, respectively. A total of 5015 candidates were retrieved from PubChem. Based on the MetFrag score and retention time model, a most probable compound (3-(2,5-dimethoxyphenyl)propanoic acid) was identified at the level of identification 3. Only two fragments (135.0470 and 209.0822) matched to the ones from the reference compound in METLIN (METLIN ID: 24106) mass spectral library. Therefore, more information is needed to increase identification confidence.

For the mass feature with *m*/*z* 389.1243, t_R_ = 3.71 min, the molecular formula of C20H22O8 was assigned with the mass accuracy and isotopic fit of −0.3 ppm and 40.3 mSigma, respectively. A total of 720 candidates were retrieved from PubChem. 3-[[3-[(3S,4R,5S,6R)-3,4,5-Trihydroxy-6-(hydroxymethyl)oxan-2-yl]phenoxy]methyl]benzoic acid was found to be the best candidate explaining the fragments (16 out of 18) and lowest retention time prediction error, and it was tentatively identified at the level of identification 3.

For the mass feature with *m*/*z* 253.0512, tR=9.66 min, the molecular formula of C_15_H_10_O_4_ was assigned with the mass accuracy and isotopic fit of −2.2 ppm and 10.4 mSigma, respectively. Chrysin was matched to this mass and was already included in the suspect list. All the fragments were explained via in silico fragmentation tools. The fragments from the reference compound in the mass spectral library (MetaboBASE0012) were also matched to the observed ions.

For the mass feature detected at *m*/*z* 283.0611, t_R_ = 10.15 min, the molecular formula of C_16_H_12_O_5_ was assigned with a mass accuracy and isotopic fit of 0.3 ppm and 29.4 mSigma, respectively. Acacetin, a compound already included in the suspect list, was matched to this marker after comparing the MS/MS fragments (in silico and reference compounds from METLIN (METLIN ID: 48899)) and retention time prediction.

For the mass feature detected at *m*/*z* 201.1135, t_R_ = 3.94 min, the molecular formula of C_10_H_18_O_4_ was assigned with the mass accuracy and isotopic fit of −3.3 ppm and 25.6 mSigma, respectively. Sebacic acid was identified tentatively after matching the observed MS/MS fragments with those in the literature (METLIN ID: 4240).

For the mass feature with *m*/*z* 315.0522, t_R_ = 7.86 min, the molecular formula of C_16_H_12_O_7_ was assigned to the observed mass with the mass accuracy and isotopic fit of −3.7 ppm and 55.1 mSigma, respectively. Isorhamnetin, from the suspect list, was matched to this mass after comparing the MS/MS fragments (in silico and reference compounds from METLIN (METLIN ID: 3445) and retention time prediction.

## 3. Materials and Methods

### 3.1. Chemicals

All the reagents and solvents used for the UPLC–QToF-MS analysis were of high analytical purity. In particular, LC–MS grade methanol (MeOH) and ACN were purchased from Merck (Darmstadt, Germany), while EtAc, sodium sulfate anhydrous and ammonium acetate (purity 99.0% or greater) were purchased from Sigma-Aldrich (Stenheim, Germany). Ultrapure water (18.2 ΜΩ cm^−1^) was provided by a Milli-Q water purification system (Direct-Q UV, Millipore, Bedford, MA, USA). Apigenin (purity ≥99%), ethyl vanillin (purity ≥98.5%), ferulic acid (purity 99%), hydroxytyrosol (purity ≥98%), luteolin (purity ≥97.0%), p-coumaric acid (purity ≥98.0%), quercetin (purity ≥95%), vanillin (purity 99%), cinnamic acid (purity 99%), eriodictyol (purity ≥98.0%), taxifolin (purity ≥85%), vanillic acid (purity ≥97.0%), syringic acid (purity ≥98%), 4-hydroxybenzoic acid (purity ≥99%), 3,4-dihydroxybenzoic acid (purity ≥97.0%), 2,5-dihydroxybenzoic acid (purity ≥99.0%), salicylic acid (purity ≥99.0%), gallic acid monohydrate (purity ≥99.0%), and caffeic acid (purity ≥98.0%) were purchased from Sigma-Aldrich while tyrosol (purity 98%) was purchased from Alpha Aesar. Finally, regenerated cellulose syringe filters (R.C. filters, pore size 0.2 μm, diameter 15mm) were acquired from Phenomenex (Torrance, CA, USA).

### 3.2. Preparation of Standards

Stock solutions of 1000 mg/L were prepared for each analyte in MeOH and they were stored at −20 °C in amber glass bottles to prevent photodegradation. Mix working solutions containing all the analytes at concentrations of 25 and 50 mg/L were prepared and stored in the refrigerator. The working solutions were appropriately diluted with MeOH:H_2_O 50:50 to construct calibration curves for all analytes at the concentrations of 0.25, 0.50, 1.0, 2.0, and 5.0 mg/L. Moreover, similar calibration curves were constructed in the blank honey matrix (matrix-matched calibration curve) to assess the method’s quality and validation parameters (linearity, precision, and matrix effects) and also for analyte quantification. The matrix-matched standards were prepared by spiking the compounds in a blank honey extract prior to injection.

### 3.3. Honey Samples

Fifty-one honey samples from ten different botanical origins, namely acacia, arbutus, blossom, buckwheat, chestnut, fir, heather, linden, rape, and thyme, were collected directly from Greek and Polish producers. The obtained samples were stored in dark glass containers at 4 °C before analysis. A detailed sample description can be found in Section S4, Table S5, of ESM. Before analysis, all samples were mixed thoroughly for 3 min to obtain a homogeneous sample. In case the honey was crystallized, it was previously heated in a water bath at no more than 40 °C until liquefying was complete. A QC sample was prepared by mixing 20 μL of each honey sample extract to obtain a pooled sample containing information from all different botanical and geographical origins. The QC sample was used during the sequence to assess potential drifts as well as during data processing to evaluate if the analysis was performed in a reliable and reproducible way. Finally, an ultrapure water sample was subjected through the whole procedure and was used as a procedural blank to subtract possible contamination during data processing.

### 3.4. UPLC–QToF-MS Analysis

The experiments were performed using an Ultra-High Performance Liquid Chromatographic system (UPLC, Dionex UltiMate 3000 RSLC, Thermo Fisher Scientific (Driesch, Germany) coupled with a Q-ToF mass spectrometer (Maxis Impact, Bruker Daltonics (Bremen, Germany). The chromatographic separation was performed on an Acclaim RSLC C18 column (2.1 × 100 mm, 2.2 μm) from Thermo Fischer Scientific, equipped with an Acquity UPLC BEH C18 VanGuard Pre-Column from Waters, and thermostated at 30 °C. The mobile phase mixtures were composed of (A) Milli-Q H_2_O:MeOH (90:10) and (B) MeOH, both A and B containing 5 mM ammonium acetate. The LC gradient elution and flow rate program is described in Table S6 of Section S5 of the ESM. The injection volume was set to 5 µL. The QToF-MS system was equipped with an ESI source, operating in negative ionization mode. The operation parameters for ESI were set as follows: capillary voltage, 3500 V; end plate offset, 500 V; nebulizer gas pressure 2 bar (N2); drying gas, 8 L min^−1^; and dry temperature, 200 °C.

The QTOF-MS system was operating in broadband collision-induced dissociation (bbCID) acquisition mode and recorded spectra over the *m*/*z* range 50−1000 with a scan rate of 2 Hz. The Bruker bbCID mode is a data-independent acquisition mode (DIA) that provides MS and MS/MS spectra at the same time, working at two different collision energies; at low collision energy (4 eV), MS spectra are acquired while at high collision energy (25 eV), MS/MS spectra are collected. Honey samples from each botanical and geographical origin, as well as the pool QC sample, were also analyzed using a data-dependent acquisition mode (DDA), AutoMS. In AutoMS, the five most abundant ions per MS scan are selected and fragmented, providing precise and compound-specific MS/MS spectra and, thus, this mode is most suitable for the structure elucidation of unknowns. In case important ions, such as that found to discriminate the geographical origin of honey samples, have not been included in the 5 most abundant ions, another AutoMS run was performed using an inclusion list containing these ions.

QTOF-MS external calibration was performed before analysis with a 10 mM sodium formate solution in a mixture of water/isopropanol (50:50). The theoretical exact masses of calibration ions with formulas HCOO(NaCOOH)_1–14_ in the range of 50−1000 Da were used for calibration. Internal calibration was also performed using a calibrant injection at the beginning of each run in a dedicated calibration segment (0.1−0.25 min).

### 3.5. Sample Preparation Optimization

A liquid–liquid extraction (LLE) protocol for the extraction of honey bioactive content was developed and optimized. Although extraction protocols using Amberlite XAD-2 resin or SPE have shown great recovery rates for the selective extraction of phenolic compounds in honey [60], LLE, using various extraction media, is a generic methodology more suitable for metabolomic studies as wider analytical information can be acquired [23]. Thus, we investigated the extraction efficiency of two solvents, EtAc and ACN, as well as a mixture of EtAc:ACN (50:50). The selection of these particular solvents was made according to the literature as they have been previously utilized in honey authenticity studies [26,31].

The selection of the most suitable extractant was performed by conducting recovery experiments in a blossom honey sample. This specific honey was chosen as it has been previously analyzed, and only traces of the tested compounds were detected. The spiked test set contained the following analytes: apigenin, ethyl vanillin, ferulic acid, hydroxytyrosol, luteolin, p-coumaric acid, quercetin, tyrosol, and vanillin. Although hydroxytyrosol and tyrosol have never been mentioned to exist in honey, they were included in the test set since they have structural similarity with some of the compounds naturally occurring in honey. For the same reason as above, ethyl vanillin, which is a synthetically produced compound, was also included in the test set. Three subsamples for each extraction procedure were weighed and spiked with the abovementioned working solution of phenolic compounds. The fortification level was at the concentration of 0.2 mg kg^−1^ (1 mg/L in the extract). The recoveries were calculated by dividing each analyte area in the spiked sample with that of a matrix-matched standard prepared at the same concentration.

In the final optimized sample preparation protocol, 1.0 g of well-homogenized honey sample was weighed and diluted in 5 mL of acidified water (pH < 2) containing 2% sodium chloride. After being vortexed for 1 min, the sample was extracted 3 times with 5 mL of EtAc. Between each extraction step, the samples were centrifuged to obtain better separation of the two phases. The organic phases were combined and dried with anhydrous sodium sulfate. The extract was evaporated under a gentle nitrogen stream to dryness and then reconstituted to 0.2 mL with a final proportion of MeOH:H_2_O (50:50). Finally, the reconstituted extracts were filtered through a 0.2 µm RC syringe filter and were ready for injection in the chromatographic system.

### 3.6. Validation

To demonstrate the feasibility of the developed methodology and test its applicability, the method’s performance was thoroughly evaluated in terms of trueness, repeatability, intermediate precision, selectivity, linearity, limits of detection (LODs) and limit of quantification (LOQs). The validation set consisted of 17 phenolic compounds, namely apigenin, ferulic acid, luteolin, p-coumaric acid, quercetin, vanillin, cinnamic acid, eriodictyol, taxifolin, vanillic acid, syringic acid, 4-hydroxybenzoic acid, 3,4-dihydroxybenzoic acid, 2,5-dihydroxybenzoic acid, salicylic acid, gallic acid, and caffeic acid which have been previously reported to exist in honey. The validation experiments were performed using the blossom honey sample used already for the method optimization experiments. A further description of the validation procedure is provided in Section S6 of the ESM.

### 3.7. Screening Approaches

Chromatography combined with low-resolution tandem mass spectrometry is commonly used for target screening of specific analytes in honey, providing a wide linear range and great sensitivity. However, the predefined target list scanned for selected reaction monitoring (SRM) can be a limitation for an authenticity study. Instead of utilizing the SRM mode, an HRMS detector, such as QToF-MS, operating in full scan mode can be a solution by generating analytical information useful both for target and non-target screening.

#### 3.7.1. Target Screening Approach

An accurate-mass target screening database was compiled and used to identify and quantify specific phenolic compounds in all honey samples. This database consisted of 21 phenolic compounds covering different classes such as phenolic acids, phenolic alcohols, phenolic aldehydes, and flavonoids. The detailed target list can be found in the ESM (Section S2, Table S3). The information included in the target list was the name of each compound, the molecular formula, the retention time and the exact masses of the precursor and qualifier ions (in-source or MS/MS fragments). The identification criteria were as follows: retention time tolerance lower than ±0.2 min, mass accuracy of the precursor and qualifier ions less than 5 mDa, isotopic fit less or equal than 50 mSigma (Bruker mSigma is a measure of the goodness of fit between the measured and the theoretical isotopic pattern) and the existence of at least two qualifier ions. The target screening was performed using the software TASQ 1.4 and DataAnalysis 4.4 (Bruker Daltonics, Bremen, Germany) along with other tools included in this software, such as Bruker Compass Isotope Pattern and SmartFormula Manually.

The quantification of identified target analytes was performed using matrix-matched calibration curves (see Section 3.2). The concentration of each analyte was determined using the corresponding equation from the matrix-matched calibration curve. Matrix-matched calibration curves were used instead of external standard calibration curves in order to compensate for ion suppression or enhancement phenomena and, thus, a more precise assessment of the concentration to be achieved.

#### 3.7.2. Suspect Screening Approach

Suspect and non-target screening workflows were applied as suggested by Krauss et al. [61] and Gago-Ferrero et al. [62]. Initially, an in-house suspect list of compounds encountered in honey was composed. The suspect list consisted of 60 bioactive constituents, which have been previously reported in the literature to exist in honey samples of different botanical and geographical origins [2,26,36,48,49,54,55,58,63,64,65,66,67,68,69,70,71,72,73]. The information included in the suspect database was the chemical name of the compounds, their molecular formula, the monoisotopic mass and pseudomolecular ion, possible adducts and fragments ions as well as the predicted retention time calculated by an in-house retention time prediction model based on the quantitative structure retention relationship (QSRR) [39]. The suspect database compiled and used to screen the honey samples is presented in the ESM (Section S7, Table S7). The suspect screening workflow and the parameters used for the identification of the suspect compounds have been thoroughly described previously by our group [74]. Briefly, the extracted ion chromatograms of pseudomolecular ions of the suspect compounds were created by the TASQ 1.4 software using the following parameters: mass accuracy threshold of 5 mDa, isotopic fit below or equal to 50 mSigma, ion intensity more than 1000 and peak area threshold more than 2000. In case any peak was detected, the existence of the suspect compound was confirmed by meticulous examination of the isotopic fit as well as the comparison of MS/MS fragments with that found in mass spectral libraries such as Fiehn Lab MassBank of North America [75] and METLIN [76] or by in silico fragmentation tools such as MetFrag [77]. Predicted retention time was also calculated using the quantitative structure-retention relationship model (QSRR) [78] and compared to the experimental t_R_ before MS/MS evaluation. This was to exclude potential false positives or to distinguish between structural isomers/tautomers, which often have identical MS/MS fragmentation pattern. The acceptance criteria for the predicted retention time and the model’s applicability domain were thoroughly presented in a previous work of our lab [39].

#### 3.7.3. Non-Target Screening Approach

The first step in non-target screening was to perform peak picking and compilation of a list with masses of interest. The mass of interest is generally a mass with high ionization efficiency and instrumental response factor (peak area/intensity) or a mass prioritized by chemometric methods (markers). All the raw data from UPLC–QToF-MS analysis were converted into mzXML files using the export chromatogram module provided by Data Analysis 4.4 (Bruker Daltonics, Bremen, Germany). These raw data were then imported into the R environment, and pick peaking was performed by the centWave algorithm implemented in the XCMS R package. XCMS has been widely used in LC–HRMS data processing due to its high efficiency and sensitivity in detecting potential region-of-interesting mass traces (ROIs). The three main internal parameters of XCMS were optimized using the IPO R package. These parameters were mass accuracy in ppm (which is the threshold for tolerated mass deviation), minimum and maximum chromatographic peak width and, finally, snthresh ratio that applies to the chromatographic signal-to-noise threshold to extract peaks list. In this study, the optimized values we used were 23.3, 17.5, and 40, respectively. The signal-to-noise threshold was also set to 3, and the prefilter was adjusted at 3-1000. The parameter “prefilter” is a threshold for an *m*/*z* to be considered as a true peak if it exists in k consecutive scan at J intensity threshold (k,I)). The non-linear retention time alignment wrapping algorithm by loess (rector function in XCMS) and peaks group across samples (group function in XCMS) were used to correct and align retention time drift among samples. Missing peaks across samples were filled and the annotation of extracted *m*/*z* features was done by CAMERA and non-target R package. This was highly needed to prevent adducts/isotopic peaks to cofound with their molecular ions. Consequently, a table including 51 analyzed honey samples and 408 mass features (*m*/*z*s, t_R_ RT) was generated.

After peak picking, the masses (*m*/*z*) that originated from the analytical procedural blank were subtracted from the sample peaks list using an advanced chemometric method based on deep learning [79]. The remaining peaks list or mass of interest were searched within the list of known unknowns of 259,298 natural products and corresponding candidates (within a certain mass accuracy (2mDa) were retrieved. This list is compiled from JNP (journal of natural products), GNPS, PubChem, FooDB, Super Natural II, and dictionary of natural products. This database is chemically curated to be suitable for screening using mass spectrometry [80]. Next, the theoretical isotopic pattern was calculated for each candidate (for its given formula) using the “enviPat” R package and then compared with extracted experimental isotopic pattern using dot product algorithm. Then, each candidate’s experimental and predicted retention time was compared to facilitate the identification. For interpretation of MS/MS fragments, the in silico fragmentation tools of MetFrag and CFM-ID were used to further prioritize the candidates for given masses. Finally, the public and open access mass spectral libraries (MassBank, METLIN, mzCloud and GNPS) were used to verify the identification of the compounds at the level of identification 2a [81]. The identification levels are described in detail in the ESM (Section S8). The threshold of 0.7 [62] was applied to the MS/MS similarity score between the MS/MS spectra of the candidates and the reference standards. In case of the absence of intensity information in the MS/MS spectra of the reference standard, the three least matched fragments were used to confirm the detected compound. All these steps were done by an in-house program called “AutoSuspect” [82].

### 3.8. Chemometrics

Chemometrics were used to discriminate between honey samples from Greece and Poland as well as to reveal the geographical origin markers. Prior to multivariate analysis, the peak list was scaled as it was vital to regulate the relative importance of each mass in the peaks list in the subsequent model. In the present study, different scaling methods, such as Pareto scaling, unit variance scaling, and autoscaling, were evaluated with principal component analysis (PCA) and partial least square discriminant analysis (PLS-DA), but poor classification was observed. Therefore, it was concluded that masses were confounding, and the difference between the geographical origins of honey samples is significantly obscured by spurious variation. To resolve this, the variance stability (Vast) scaling was used as the optimal pre-processing method to build a reliable supervised classification model. Vast scaling is the opposite of Pareto scaling, which increases and decreases the effect of markers that have small and large standard deviation, respectively. Consequently, the peaks list was scaled using the Vast scaling method [83,84] and PLS-DA was used as the supervised classification technique to discriminate between Polish and Greek honey samples [85]. Furthermore, the variable importance in projection (VIP) selection method was used to find potential markers behind the PLS-DA model that can discrimination between samples of different origin [86]. In brief, VIP scores capture the influence of individual *m*/*z* value, and they are calculated as the weighted sum of squares of the PLS-DA weights according to Equation (1).

(1) $$VIP_{i}=\sqrt{\frac{\sum_{c=1}^{C}w_{ic}^{2}\cdot SSY_{c}\cdot I}{SSY_{total}\cdot C}}$$

*w_ic_* is the weight value for i *m*/*z* value in the peaks list and *c* component, *SSY_c_* is the sum of squares of the explained variance in the *cth* component, *I* refers to the total numbers of *m*/*z* value in the peaks list, *SSY_total_* is the total sum of squares explained in the categorical variable (here is the geographical origin) and *C* is the total number of components. Therefore, *VIP_i_* provides the total contribution of each *m*/*z* value in the peaks list in the PLS-DA model. Because the average of the squared *VIP* scores equals 1, any *m*/*z* with *VIP* greater than 1.0 is significant in the PLS-DA model. Therefore, the cut-off value of 1.0 was used for the VIP score. All the chemometrics methods used here were implemented in an in-house toolbox, written in MATLAB, called ChemoTrAMS.

#### Validation Criteria for Multivariate Analysis

The PLS-DA model was validated internally and externally using misclassification error rate in leave-one-out cross validation and set of 11 samples as test set, respectively. These 11 samples were not part of the initial training set, and they were selected using the Kennard–Stone method [87] as described in a previous work of our group [88]. The total error rate, class specificity, and sensitivity, as well as class assigned probabilities, were calculated and used in receiver operating characteristics (ROC) curve. A classification model is internally well established if it gives a point in the upper left corner of the ROC curve, representing the total area under curve equal to 1 as well as maximum sensitivity and specificity, while a random model shows a curve that locates around the diagonal line from the left bottom to the top right corner [89].

## 4. Conclusions

Targeted and non-targeted metabolomic workflows were developed and applied using a UPLC–QToF-MS method. Simultaneous determination of phenolic compounds and geographical origin discrimination were achieved in 10 different honey matrices (*n* = 51) originating from Greece and Poland. To accomplish that, a generic LLE protocol was applied using EtAc as the extractant. Quality performance characteristics were evaluated, indicating an accurate, sensitive, and precise method. In terms of targeted analysis, 21 compounds were used for screening purposes, and among them, p-coumaric acid was the most abundant in 3 different matrices (linden, rape, and thyme) whilst 4-hydroxybenzoic acid was found in high concentration in buckwheat honey. Through suspect screening, 16 compounds were identified in at least one sample. The identified abscisic acid isomers, namely 2-*cis*,4-*trans*-abscisic acid and 2-*trans*,4-*trans*-abscisic acid as well as homogentisic acid, were the most abundant in arbutus honey. Moreover, dehydrovomifoliol and phenyllactic acid were higher in Polish heather honey than Greek, while methyl syringate was found most abundant in Rape honey. Importantly, 10 markers were identified, 6 for Greek and 4 for Polish honey, using a non-targeted workflow. The marker identification level varied from 2a to 4, indicating the necessity to apply comprehensive chemometric tools to reveal authenticity markers. The developed method highlights the analytical capabilities provided by HRMS and the importance of determining phenolic compounds in honey for their dietary impact and exploitation as authenticity markers.

## Figures and Tables

**Table 1:** Phenolics concentration expressed as a median value in various unifloral honeys.

Compound	Median Concentrations (mg/kg)									
	Acacia (*n* = 6)	Arbutus (*n* = 2)	Blossom (*n* = 2)	Buckwheat (*n* = 8)	Chestnut (*n* = 2)	Fir (*n* = 2)	Heather (*n* = 10)	Linden (*n* = 9)	Rape (*n* = 7)	Thyme (*n* = 3)
Apigenin	0.14	<LOD	<LOD	0.13	<LOD	<LOD	0.36	0.13	<LOD	<LOD
Cinnamic acid	0.061	0.050	0.54	0.21	0.61	<LOD	2.1	0.10	0.052	0.049
Ferulic acid	0.83	0.051	0.14	0.18	0.18	0.032	0.15	0.24	0.36	0.061
Luteolin	<LOD	<LOD	0.18	<LOD	<LOD	<LOD	<LOD	0.11	ND	0.083
p-Coumaric acid	0.22	<LOD	1.0	4.2	1.7	0.45	0.29	1.5	1.1	1.3
Quercetin	<LOD	0.19	<LOD	0.13	<LOD	5.0	0.18	0.15	<LOD	0.13
Salicylic acid	0.40	<LOD	1.2	1.8	ND	0.56	ND	0.88	0.74	<LOD
Taxifolin	ND	ND	0.51	<LOD	0.15	0.61	<LOD	ND	<LOD	0.16
Vanillin	0.27	<LOD	0.54	0.37	ND	0.11	0.25	0.20	0.26	0.089
3,4-Dihydroxybenzoic acid	0.13	0.57	0.50	0.41	2.9	2.5	0.28	0.54	0.11	0.47
4-Hydroxybenzoic acid	0.50	2.9	0.94	15	1.3	1.3	2.1	1.1	1.0	0.60

**Table 2:** Identified compounds through suspect screening.

Compound	Molecular Formula	*m*/*z* (Pseudo-Molecular Ion)	Mass Error (mDa)	t_R_ Experimental (min)	t_R_ Predicted (min)	Isotopic Fitting (mSigma)	*m*/*z* (Fragment Ions)	No of Samples Detected (in Total 51 Samples)
**2-*trans*,4-*trans*-Abscisic acid**	C_15_H_20_O_4_	263.1289	0.3	4.02	4.44	15	219.1396; 204.1159	51
**2-*cis*,4-*trans*-Abscisic acid**	C_15_H_20_O_4_	263.1289	0.5	4.45	4.44	15.7	153.0922; 219.1396; 204.1159	51
**Acacetin**	C_16_H_12_O_5_	283.0612	0.3	9.87	8.09	17.5	268.038	42
**Chrysin**	C_15_H_10_O_4_	253.0506	0.1	9.14	8.15	14.3	143.0502; 209.0611	51
**Dehydrovomifoliol**	C_13_H_18_O_3_	221.1183	0.3	5.07	5.42	17.6	206.0948; 179.1078; 161.0972; 108.0217; 191.0714	38
**Galangin**	C_15_H_10_O_5_	269.0455	0.1	10.02	7.48	16.5	239.0349; 227.0349; 223.0404	51
**Homogentisic acid**	C_8_H_8_O_4_	167.0350	0.6	1.68	3.29	20.8	122.0383; 108.0221	14
**Isorhamnetin**	C_16_H_12_O_7_	315.0510	0.2	7.96	7.63	24.6	300.0277	51
**Kaempferol**	C_15_H_10_O_6_	285.0405	0.9	8.17	7.26	7.4	229.051	45
**Lumichrome**	C_12_H_9_N_4_O_2_	241.0731	0.4	6.42	5.85	22.3	198.0675; 170.0739	40
**Methyl Syringate**	C_10_H_12_O_5_	211.0612	0.4	5.95	5.77	21.6	181.0144; 153.0194	43
**Phenyllactic acid**	C_8_H_8_O_2_	165.0557	0.6	3.13	3.65	5.8	119.0506; 147.0455; 72.9935; 103.0558	47
**Pinobanksin**	C_15_H_12_O_5_	271.0612	0.4	7.15	7.35	4.3	253.0511; 125.0244; 197.0608	51
**Pinocembrin**	C_15_H_12_O_4_	255.0663	0.9	9.13	8.14	5.7	213.0562; 151.0037; 107.0139	51
**Rosmarinic acid**	C_18_H_16_O_8_	359.0772	0.3	4.09	5.49	34.1	161.0242; 197.0449; 179.0346	1
**Sakuranetin**	C_16_H_14_O_5_	285.0768	0.4	9.25	8.06	11.2	119.0500; 165.0191	51

**Table 3:** List of the markers detected in Greek and Polish honey.

Compound Name	Measured *m*/*z*	Molecular Formula	Exp. t_R_ (Pred. t_R_) min	Marker in Greek/Polish Honey	Level of Identification Confidence	MS/MS Fragments (5 Most Abundant Fragments) in UPLC–QTOF-MS
Quinic acid	191.0566	C_7_H_12_O_6_	1.30 (1.37)	Greek	2a	171.0319, 191.0556, 85.0298, 93.0350, 59.0146
Gentisic acid	153.0198	C_7_H_6_O_4_	1.78 (2.41)	Greek	2a	108.0214, 109.0295, 91.0189, 81.0350, 110.0326
Unknown	239.1291	C_13_H_20_O_4_	4.21	Greek	4	57.0355, 58.0392, 59.0151, 61.9911, 69.0351
Unknown	313.1803	C_20_H_26_O_3_	10.75	Greek	4	255.1954, 313.1810, 315.2538
3-(2,5-Dimethoxyphenyl) propanoic acid	209.0822	C_11_H_14_O_4_	3.49 (4.94)	Greek	3	91.0556, 72.994, 135.047, 119.0493, 147.0464
3-[[3-[(3S,4R,5S,6R)-3,4,5-Trihydroxy-6-(hydroxymethyl)oxan-2-yl]phenoxy]methyl]benzoic acid	389.1243	C_20_H_22_O_8_	3.71 (4.34)	Greek	3	165.0561, 134.0375, 193.0511, 150.0321, 178.0276
Chrysin	253.0512	C_15_H_10_O_4_	9.66 (8.05)	Polish	2a	253.0512, 143.0500, 209.0626, 63.0249, 119.0513
Acacetin	283.0611	C_16_H_12_O_5_	10.15 (8.03)	Polish	2a	268.0383, 239.0353, 211.0404, 283.0611, 167.0505
Sebacic acid	201.1135	C_10_H_18_O_4_	3.94 (4.21)	Polish	2a	139.1127, 59.0148, 165.0188, 183.1011, 121.0289
Isorhamnetin	315.0522	C_16_H_12_O_7_	7.86 (7.56)	Polish	2a	300.0275, 315.0511, 165.9912

## Data Availability

Data are available upon request to the authors.

## Supplementary Materials

Section S1: Method Development and Validation Data, Table S1: Recovery rate (% R) and SD (*n* = 3) for each spiked compound in the three different extractants tested. Table S2: Validation data of target screening methodology, Section S2: Target screening Database. Table S3: Target list of phenolic compounds, Section S3: Suspect screening Results. Figure S1: Identification data for the mass feature *m*/*z* 283.0612_9.87 min (acacetin). Figure S2: Identification data for the mass feature *m*/*z* 253.0506_9.61 min (chrysin). Figure S3: Identification data for the mass feature *m*/*z* 221.1183_5.07 min (dehydrovomifoliol). Figure S4: Identification data for the mass feature *m*/*z* 269.0455_10.04 min (galangin). Figure S5: Identification data for the mass feature *m*/*z* 167.0350 _1.68 min (homogentisic acid). Figure S6: Identification data for the mass feature *m*/*z* 315.0510_7.96 min (isorhamnetin). Figure S7: Identification data for the mass feature *m*/*z* 285.0405_8.17 min (kaempferol). Figure S8: Identification data for the mass feature *m*/*z* 241.0731_6.42 min (lumichrome). Figure S9: Identification data for the mass feature *m*/*z* 211.0612_5.96 min (methyl syringate). Figure S10: Identification data for the mass feature *m*/*z* 165.0557_3.13 min (DL-β-phenyllactic acid). Figure S11: Identification data for the mass feature *m*/*z* 271.0612_7.15 min (pinobanksin). Figure S12: Identification data for the mass feature *m*/*z* 255.0663_9.13 min (pinocembrin). Figure S13: Identification data for the mass feature *m*/*z* 359.0772_4.09 min (rosmarinic acid). Figure S14: Identification data for the mass feature *m*/*z* 285.0768_9.25 min (sakuranetin). Table S4: Number of samples of each botanical origin which have been identified each compound, Figure S15: Batch statistics graph showing the mean area and the standard deviation for dehydrovomifoliol. Figure S16: Batch statistics graph showing the mean area and the standard deviation for phenyllactic acid. Figure S17: Batch statistics graph showing the mean area and the standard deviation for Homogentisic acid, Figure S18: Batch statistics graph showing the mean area and the standard deviation for methyl syringate, Section S4: Honey Samples. Table S5: Honey sample characterization, Section S5: LC elution program. Table S6: LC gradient elution and flow rate program, Section S6: Validation procedure, Section S7: Suspect database. Table S7: Suspect list of bioactive compounds encountered in honey. Section S8: Level of identification confidence.
