# Supplementary material for: Honey Phenolic Compound Profiling and Authenticity Assessment Using HRMS Targeted and Untargeted Metabolomics
Source: Molecules. 2021 May 8;26(9):2769. doi: 10.3390/molecules26092769 (PMC8125859; doi:10.3390/molecules26092769)
Supplement: Supplementary file 1 [file molecules-26-02769-s001.zip › molecules-1210012-supplementary.pdf]

ELECTRONIC SUPPLEMENTARY MATERIAL

Honey phenolic compound profiling and authenticity assessment using HRMS targeted and untargeted metabolomics

Georgios A. Koulis, Aristeidis S. Tsagkaris, Reza Aalizadeh, Marilena E. Dasenaki*, Eleni I. Panagopoulou, Spyros Drivelos, Michał Halagarda, Constantions A. Georgiou, Charalampos Proestos, Nikolaos S. Thomaidis

Electronic Supplementary Material

Section S1: Method Development and Validation Data 4

Table S1. Recovery rate (% R) and SD (n=3) for each spiked compound in the three different extractants tested 4

Table S2. Validation data of target screening methodology 4

Section S2: Target screening Database 5

Table S3. Target list of phenolic compounds 5

Section S3: Suspect screening Results 6

Figure S1. Identification data for the mass feature m/z 283.0612_9.87 min (Acacetin). 7

Figure S2. Identification data for the mass feature m/z 253.0506_9.61 min (Chrysin). 8

Figure S3. Identification data for the mass feature m/z 221.1183_5.07 min (Dehydrovomifoliol). 9

Figure S4. Identification data for the mass feature m/z 269.0455_10.04 min (Galangin). 11

Figure S5. Identification data for the mass feature m/z 167.0350 _1.68 min (Homogentisic acid). 12

Figure S6. Identification data for the mass feature m/z 315.0510_7.96 min (isorhamnetin). 14

Figure S7. Identification data for the mass feature m/z 285.0405_8.17 min (kaempferol). 15

Figure S8. Identification data for the mass feature m/z 241.0731_6.42 min (Lumichrome). 16

Figure S9. Identification data for the mass feature m/z 211.0612_5.96 min (Methyl Syringate). 17

Figure S10. Identification data for the mass feature m/z 165.0557_3.13 min (DL-β-Phenyllactic acid). 19

Figure S11. Identification data for the mass feature m/z 271.0612_7.15 min (Pinobanksin). 20

Figure S12. Identification data for the mass feature m/z 255.0663_9.13 min (Pinocembrin). 21

Figure S13. Identification data for the mass feature m/z 359.0772_4.09 min (Rosmarinic Acid). 23

Figure S14. Identification data for the mass feature m/z 285.0768_9.25 min (Sakuranetin). 24

Table S4. Number of samples of each botanical origin which have been identified each compound 25

Figure S15. Batch statistics graph showing the mean area and the standard deviation for Dehydrovomifoliol 25

Figure S16. Batch statistics graph showing the mean area and the standard deviation for Phenyllactic acid 26

Figure S17. Batch statistics graph showing the mean area and the standard deviation for Homogentisic acid 26

Figure S18. Batch statistics graph showing the mean area and the standard deviation for Methyl syringate 26

Section S4: Honey Samples 27

Table S5. Honey samples characterization 27

Section S5: LC elution program 28

Table S6. LC gradient elution and flow rate program ... 28
Section S6: Validation procedure ... 28
Section S7: Suspect database ... 30
Table S7. Suspect list of bioactive compounds encountered in honey ... 30
Section S8: Level of identification confidence ... 33

Section S1: Method Development and Validation Data

Table S1. Recovery rate (% R) and SD (n=3) for each spiked compound in the three different extractants tested

compound	EtAc (± SD)	ACN (± SD)	EtAc : ACN (± SD)
Apigenin	102 ± 6.2	104 ± 4.5	50 ± 4.1
Ethyl vanillin	100 ± 4.5	95 ± 2.1	47 ± 5.0
Ferulic acid	104 ± 5.4	65 ± 7.3	70 ± 7.8
Hydroxytyrosol	99 ± 4.2	95 ± 5.1	74 ± 4.5
Luteolin	97 ± 6.1	97 ± 7.9	49 ± 5.7
p-coumaric acid	100 ± 4.1	54 ± 4.9	68 ± 5.1
Quercetin	98 ± 6.6	97 ± 4.9	39 ± 4.9
Tyrosol	101 ± 3.7	103 ± 5.7	72 ± 2.9
Vanillin	99 ± 4.8	88 ± 5.7	56± 4.9

Table S2. Validation data of target screening methodology

Compound	Trueness % Recovery	Repeatability %RSD$_r$ (n=6)	Intermediate precision %RSD$_R$ (n=18)	ME%	LOD mg / Kg	LOQ mg / Kg	R^2
Apigenin	94	1.8	5.6	3.6	0.082	0.24	0.990
Ferulic acid	98	3.2	6.6	6.4	0.030	0.091	0.999
Luteolin	92	11	8.7	1.5	0.079	0.24	0.991
p-coumaric acid	95	1.5	8.0	6.1	0.16	0.49	0.990
Quercetin	100	4.5	19	-8.5	0.067	0.20	0.993
Vanillin	98	3.3	8.0	-0.7	0.037	0.11	0.998
Cinnamic acid	101	5.4	4.6	-6.9	0.043	0.13	0.997
Eriodictyol	94	3.4	9.6	11	0.048	0.14	0.997
Taxifolin	77	2.0	6.7	24	0.084	0.25	0.990
Vanillic acid	78	3.8	4.4	18	0.12	0.36	0.98
Syringic acid	88	4.8	8.5	8.1	0.081	0.24	0.991
4-hydroxybenzoic acid	78	3.3	8.2	32	0.098	0.23	0.991
3,4-dihydroxybenzoic acid	74	2.8	4.3	31	0.083	0.25	0.990
2,5-dihydroxybenzoic acid	94	5.0	7.5	14	0.070	0.21	0.993
Salicylic acid	72	8.9	8.8	15	0.33	0.99	0.98
Gallic acid	76	2.0	9.4	33	0.067	0.20	0.994
Caffeic acid	79	6.2	8.0	32	0.065	0.19	0.994

Section S2: Target screening Database

Table S3. Target list of phenolic compounds

Compound	Molecular formula	$[M-H]^-$ m/z	tR (min)	q1 m/z	q1 formula	q2 m/z	q2 formula	q3 m/z	q3 formula
2,5-dihydroxybenzoic acid (gentistic acid)	$C_7H_6O_4$	153.0193	1.9	108.0215	$C_6H_4O_2$	109.0278	$C_6H_5O_2$		
3,4- dihydroxybenzoic acid (Protocatechuic acid)	$C_7H_6O_4$	153.0193	1.3	109.0294	$C_6H_5O_2$	108.0218	$C_6H_4O_2$		
4-hydroxybenzoic acid	$C_7H_6O_3$	137.0244	1.4	93.0342	C_6H_5O	65.0398	C_5H_5		
Apigenin	$C_{15}H_{10}O_5$	269.0455	8.2	269.0455	$C_{15}H_9O_5$	117.0340	C_8H_5O	151.0031	$C_7H_3O_4$
Caffeic acid	$C_9H_8O_4$	179.0349	1.4	135.0453	$C_8H_7O_2$	134.0346	$C_8H_6O_2$		
Cinnamic acid	$C_9H_8O_2$	147.0452	4.1	103.0553	C_8H_7	147.0446	$C_9H_7O_2$		
Epicatechin	$C_{15}H_{14}O_6$	289.0718	4.3	123.0452	$C_7H_7O_2$	151.0401	$C_8H_7O_3$	137.0244	$C_7H_5O_3$
Eriodictyol	$C_{15}H_{12}O_6$	287.0561	6.3	151.0038	$C_7H_3O_4$	135.045	$C_8H_7O_2$		
Ethyl vanillin	$C_9H_{10}O_3$	165.0557	5.4	136.0156	$C_7H_4O_3$	137.0222	$C_7H_5O_3$	108.0219	$C_6H_4O_2$
Ferulic acid	$C_{10}H_{10}O_4$	193.0506	2.7	134.0361	$C_8H_6O_2$	178.026	$C_9H_6O_4$		
Gallic acid	$C_7H_6O_5$	169.0142	1.2	125.0244	$C_6H_5O_3$	69.0344	C_4H_5O	97.0295	$C_5H_5O_2$
Hydroxytyrosol	$C_8H_{10}O_3$	153.0557	3.5	123.0452	$C_7H_7O_2$				
Luteolin	$C_{15}H_{10}O_6$	285.0405	7.4	285.0405	$C_{15}H_9O_6$	133.0287	$C_8H_5O_2$		
p-coumaric acid	$C_9H_8O_3$	163.0401	2.5	119.0502	C_8H_7O	93.0344	C_6H_5O		
Quercetin	$C_{15}H_{10}O_7$	301.0354	7.1	151.0036	$C_7H_3O_4$	301.0354	$C_{15}H_9O_7$	121.0288	$C_7H_5O_2$
salicylic acid	$C_7H_6O_3$	137.0244	3.2	93.0340	C_6H_5O	65.0399	C_5H_5		
Syringic acid	$C_9H_{10}O_5$	197.0455	1.4	123.0080	$C_6H_3O_3$	166.9976	$C_7H_3O_5$		
Taxifolin	$C_{15}H_{12}O_7$	303.0510	4.7	125.0227	$C_6H_5O_3$	285.0408	$C_{15}H_9O_6$	153.0193	$C_7H_5O_4$
Tyrosol	$C_8H_{10}O_2$	137.0608	4.1	137.0608	$C_8H_9O_2$	119.0495	C_8H_7O	93.034	C_6H_5O
Vanillic acid	$C_8H_8O_4$	167.0350	1.4	125.0244	$C_6H_5O_3$	152.0115	$C_7H_4O_4$		
Vanillin	$C_8H_8O_3$	151.0401	4.6	136.0158	$C_7H_4O_3$	108.0217	$C_6H_4O_2$		

Section S3: Suspect screening Results

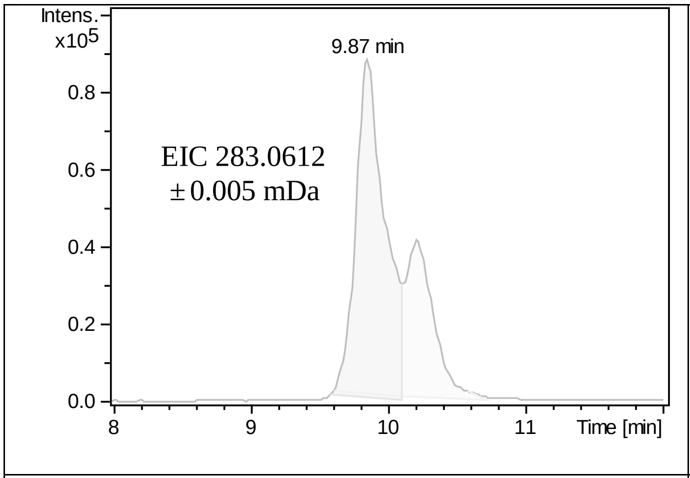

Fig S1a. EIC of m/z 283.0612 in a heather Honey

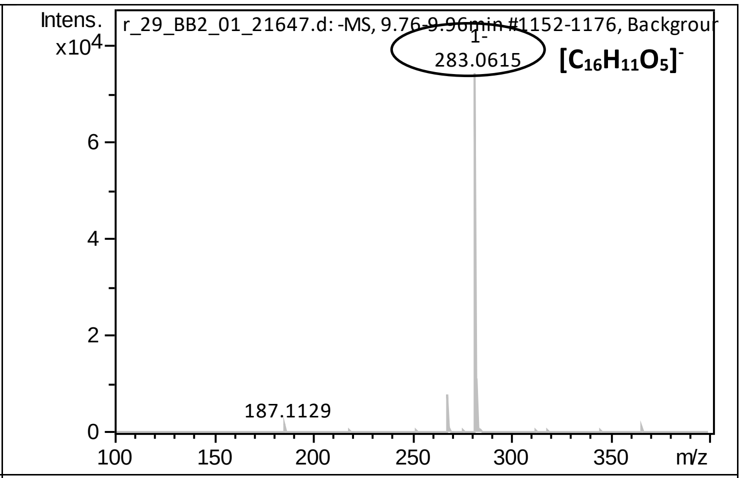

Fig S1b. Background subtracted MS Spectra from 9.7 to 10.0 min

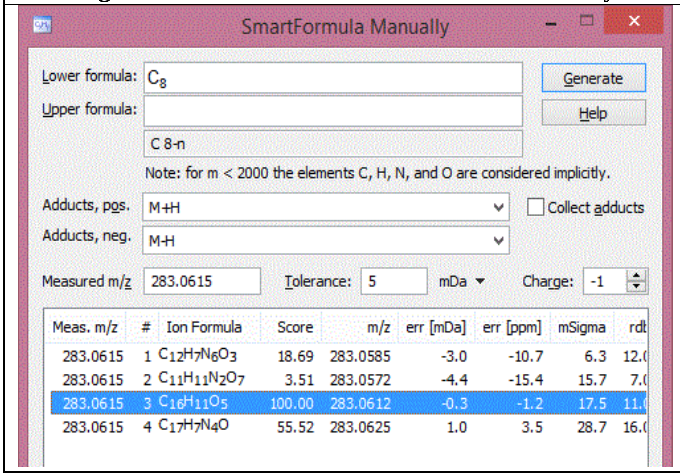

Fig S1c. Molecular Formula Annotation of m/z 283.0612

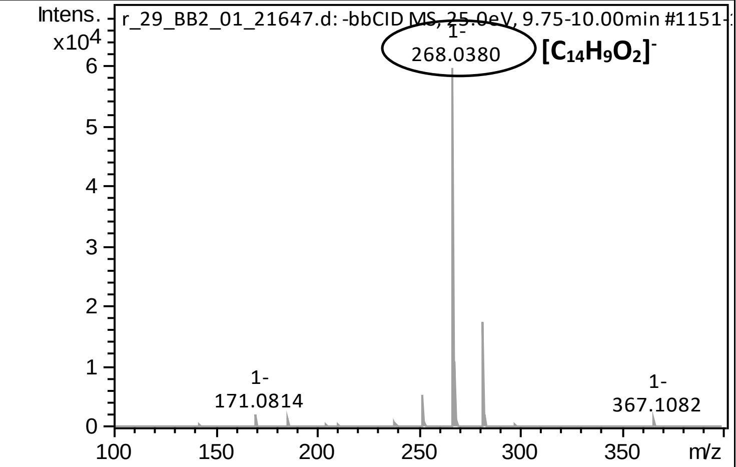

Fig S1d. Background subtracted MS/MS Spectra from 9.7 to 10.0 min

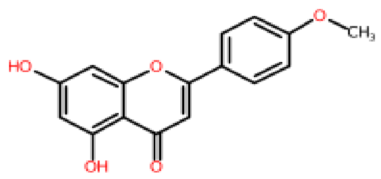

$C_{16}H_{12}O_5$

Acacetin

Precursor ion

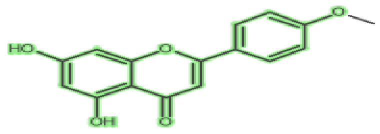

$[C_{15}H_8O_5]^-$

Mass error: 0.3 mDa

Fragment ions

Fig S1e. Structures of precursor and fragment ions of Acacetin

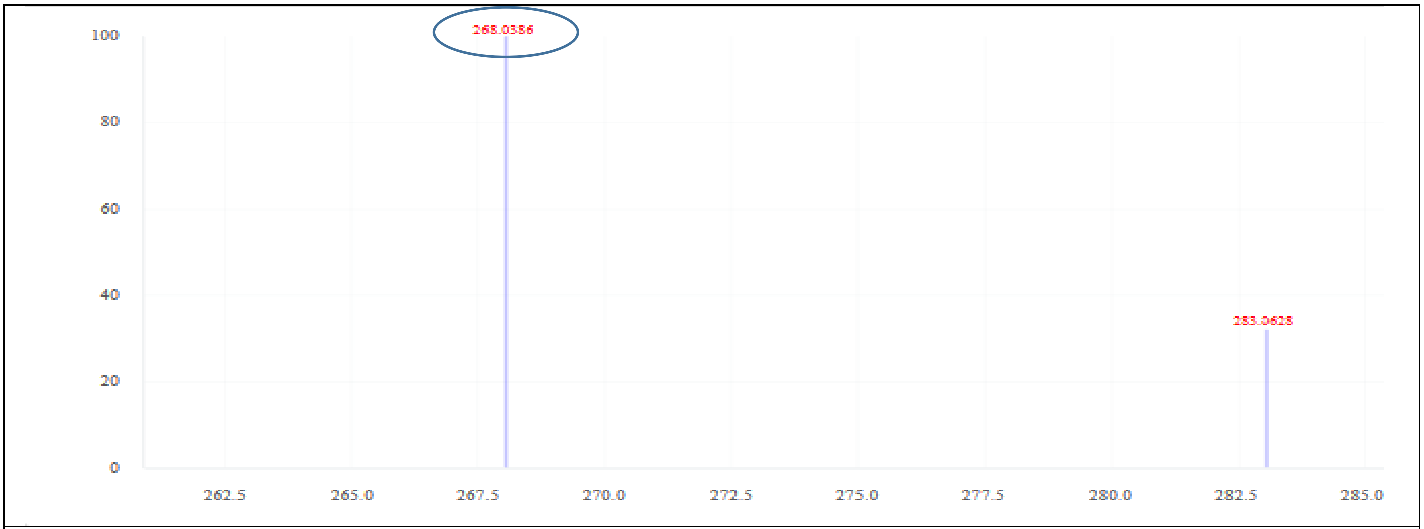

Fig. S1f: Vaniya/Fiehn Natural Products Library Record VF-NPL-QTOF006460 (Acacetin)

Figure S1. Identification data for the mass feature m/z 283.0612_9.87 min (Acacetin).

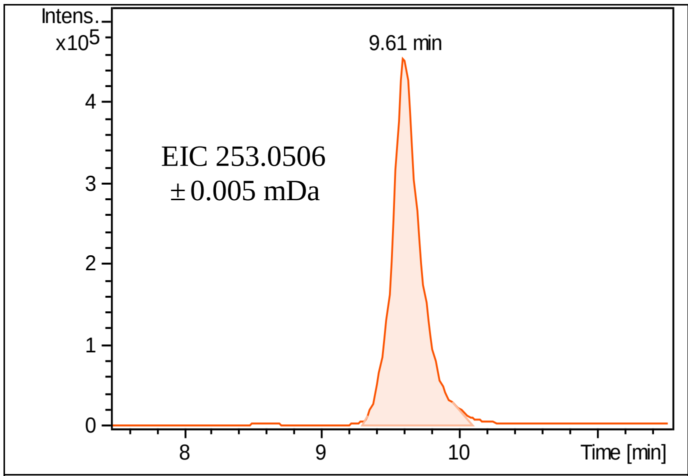

Fig S2a. EIC of m/z 253.0506 in a buckwheat Honey

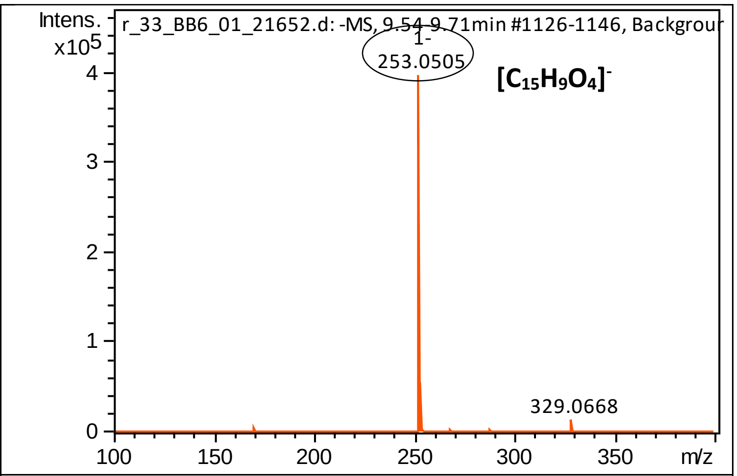

Fig S2b. Background subtracted MS Spectra from 9.5 to 9.7 min

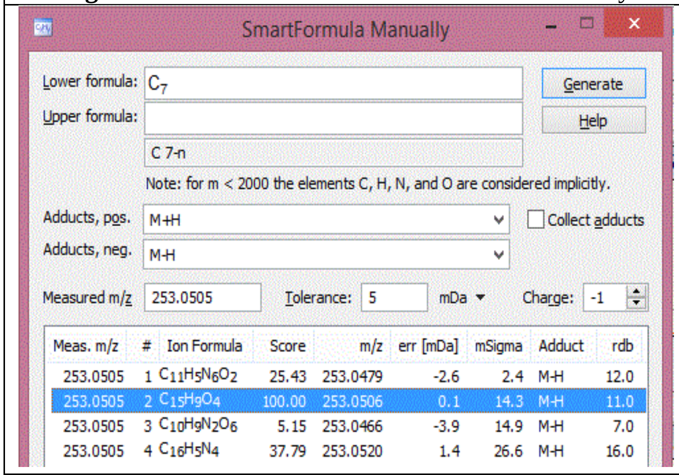

Fig S2c. Molecular Formula Annotation of m/z 253.0506

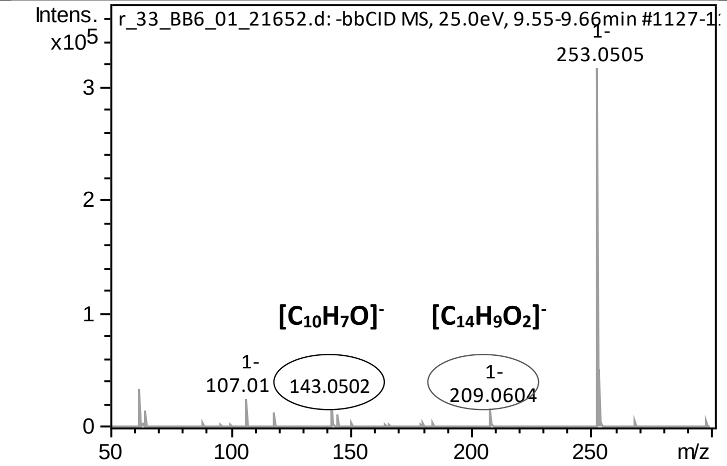

Fig S2d. Background subtracted MS/MS Spectra from 9.5 to 9.7 min

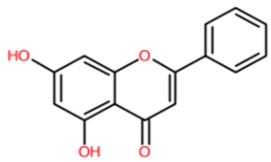

$C_{15}H_{10}O_4$
Chrysin

Precursor ion

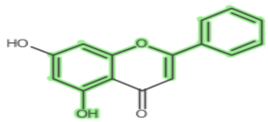

$[C_{14}H_9O_2]^-$
Mass error: 0.3 mDa

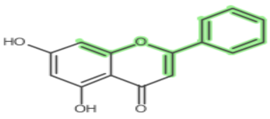

$[C_{10}H_7O]^-$
Mass error: 0.2 mDa

Fragment ions

Fig S2e. Structures of precursor and fragment ions of Chrysin

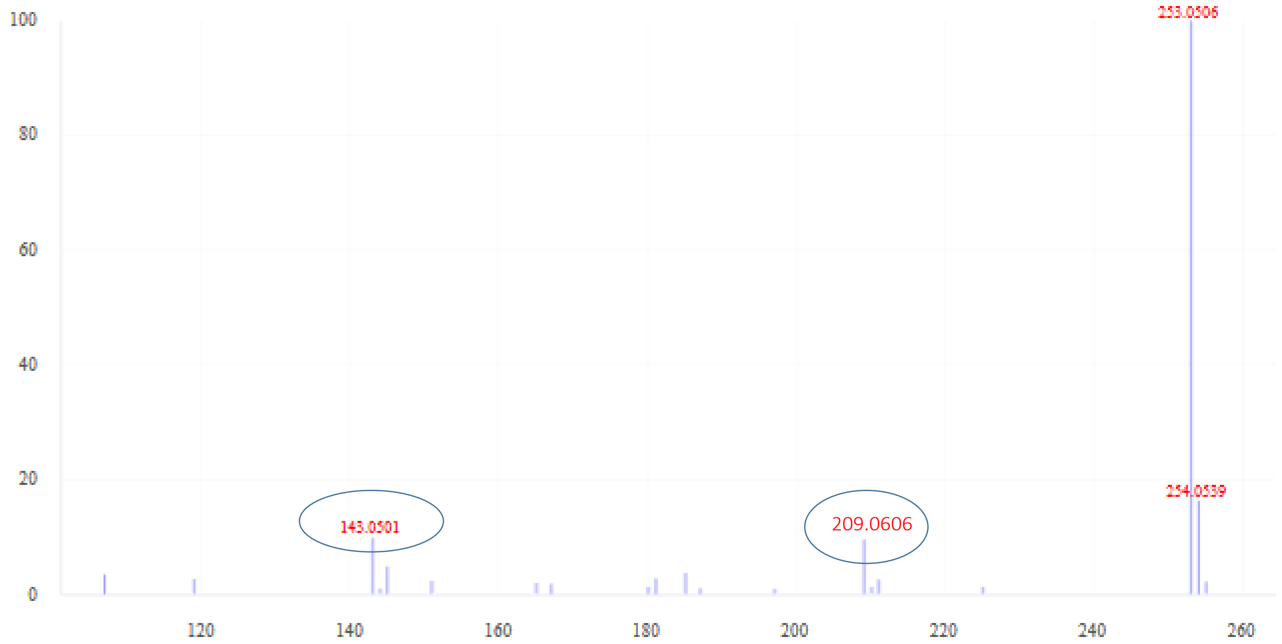

Fig. S2f: MassBank High Quality Mass Spectral Database Record FIO00030 (Chrysin)

Figure S2. Identification data for the mass feature m/z 253.0506_9.61 min (Chrysin).

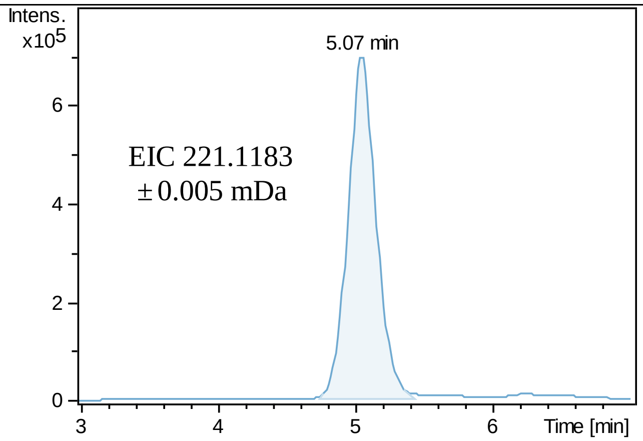

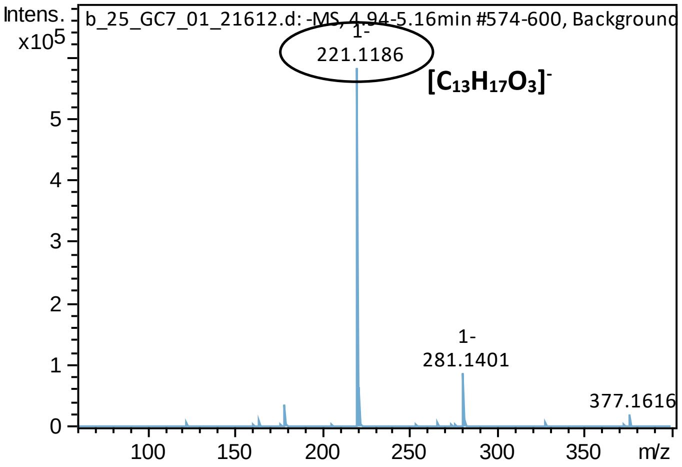

Fig S3a. EIC of m/z 221.1183 in heather Honey

Fig S3b. Background subtracted MS Spectra from 4.9 to 5.1 min

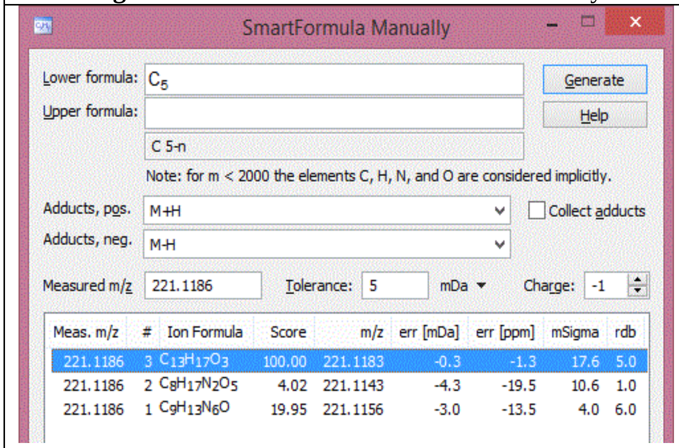

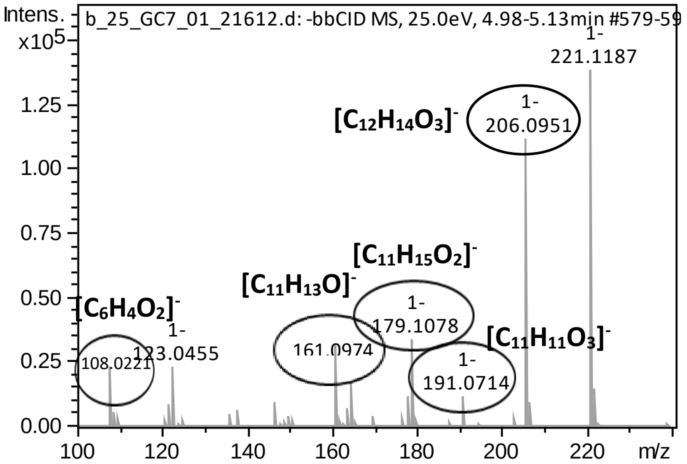

Fig S3c. Molecular Formula Annotation of m/z 221.1183

Fig S3d. Background subtracted MS/MS Spectra from 4.9 to 5.1 min

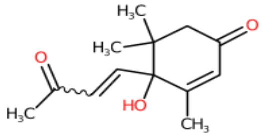

$C_{13}H_{18}O_3$

Dehydrovomifoliol

Precursor ion

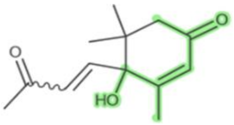

$[C_6H_4O_2]^-$
Mass error: 0.5 mDa

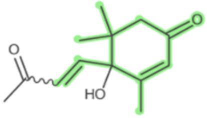

$[C_{11}H_{13}O]^-$
Mass error: 0.2 mDa

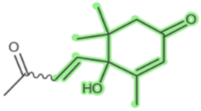

$[C_{11}H_{15}O_2]^-$
Mass error: 0.1 mDa

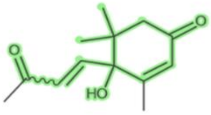

$[C_{11}H_{11}O_3]^-$
Mass error: 0.1 mDa

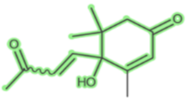

$[C_{12}H_{14}O_3]^-$
Mass error: 0.3 mDa

Fragments ion

Fig S3e. Structures of precursor and fragment ions of Dehydrovomifoliol

Figure S3. Identification data for the mass feature m/z 221.1183_5.07 min (Dehydrovomifoliol).

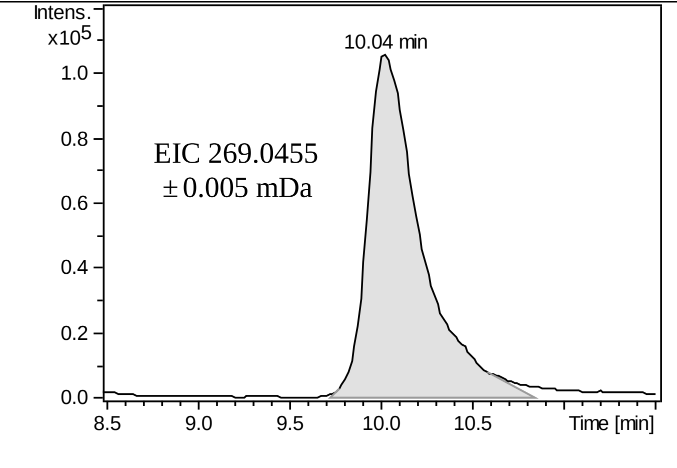

Fig S4a. EIC of m/z 269.0455in a buckwheat Honey

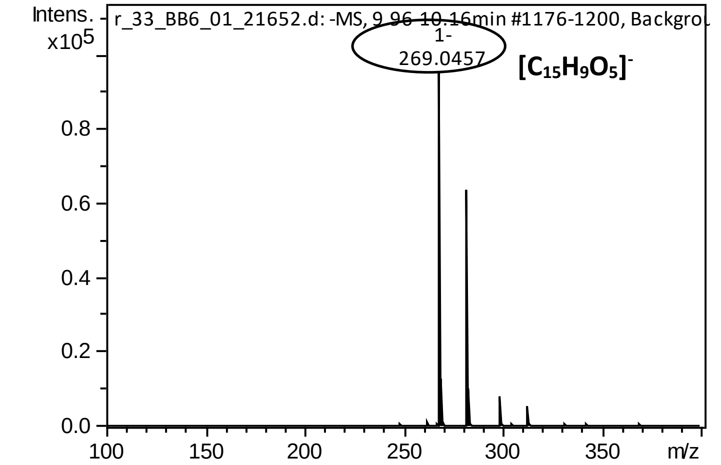

Fig S4b. Background subtracted MS Spectra from 9.9 to 10.1 min

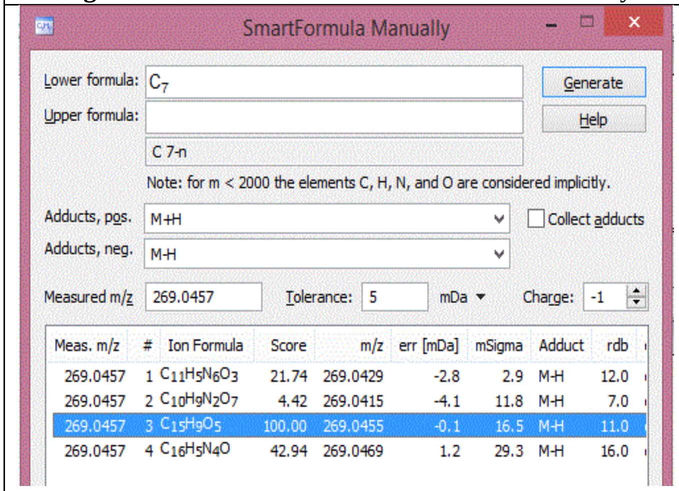

Fig S4c. Molecular Formula Annotation of m/z 269.0455

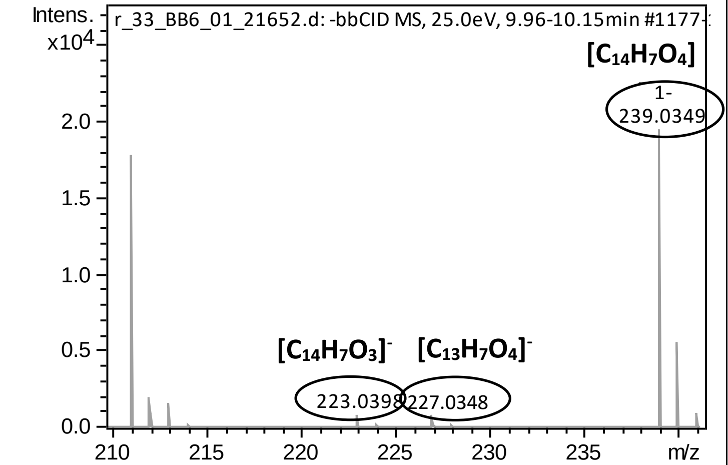

Fig S4d. Background subtracted MS/MS Spectra from 9.9 to 10.1 min

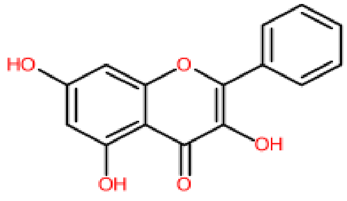

$C_{15}H_{10}O_5$

Galangin

Precursor ion

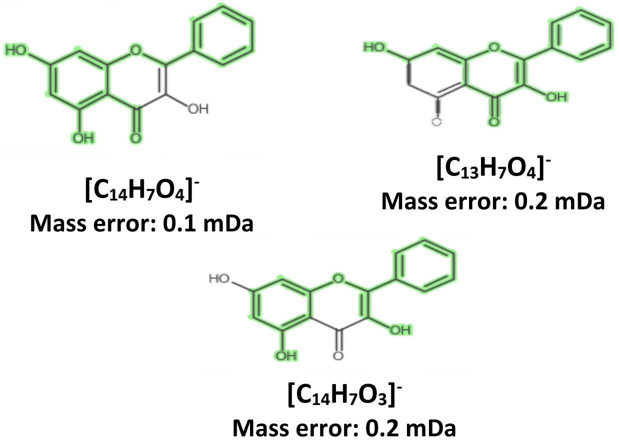

Fragment ions

Fig S4e. Structures of precursor and fragment ions of Galangin

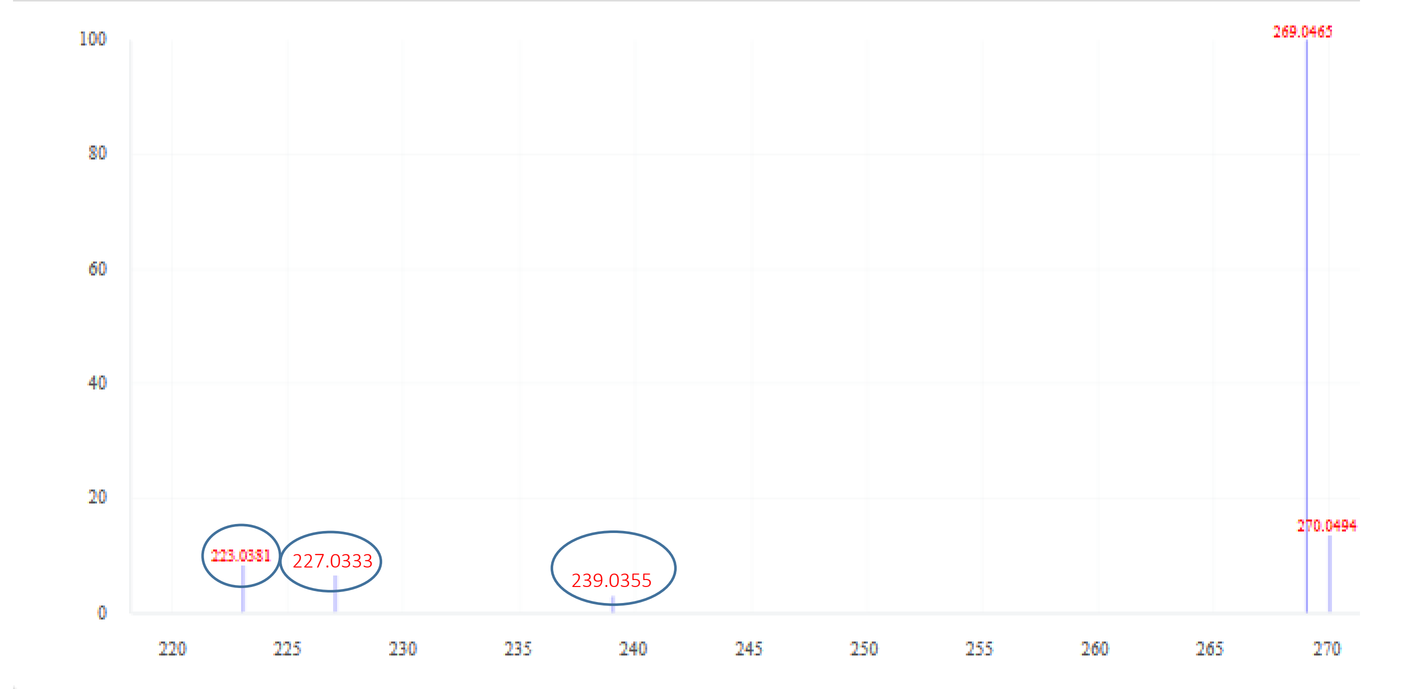

Fig. S4f: Bruker Sumner MetaboBASE Plant Library Record MetaboBASE0697 (Galangin)

Figure S4. Identification data for the mass feature m/z 269.0455_10.04 min (Galangin).

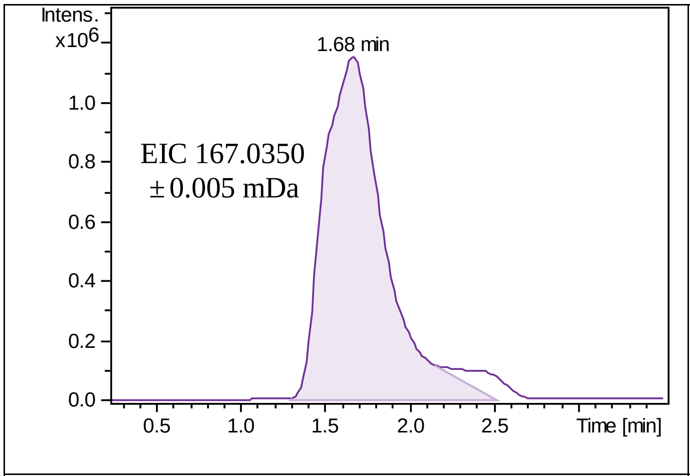

Fig S5a. EIC of m/z 167.0350 in an arbutus Honey

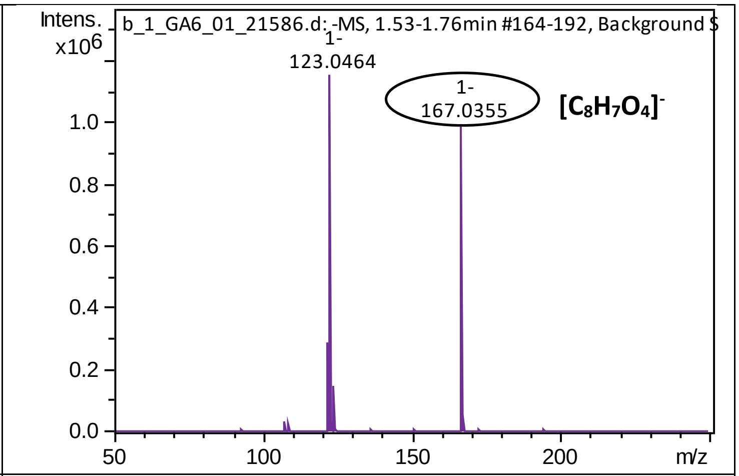

Fig S5b. Background subtracted MS Spectra from 1.5 to 1.8 min

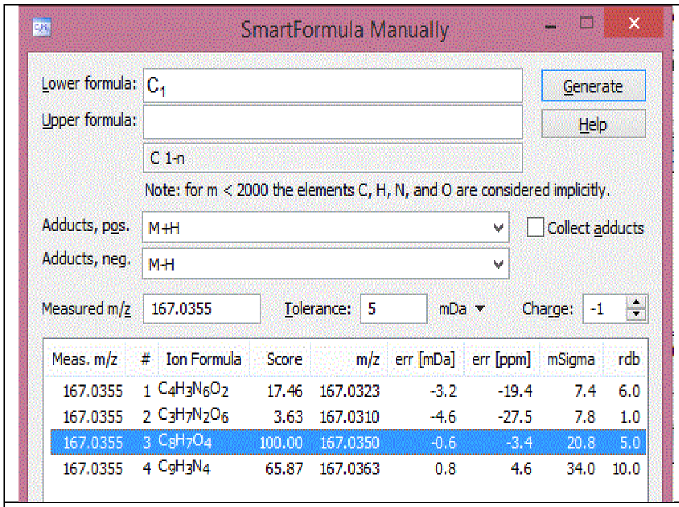

Fig S5c. Molecular Formula Annotation of m/z 167.0350

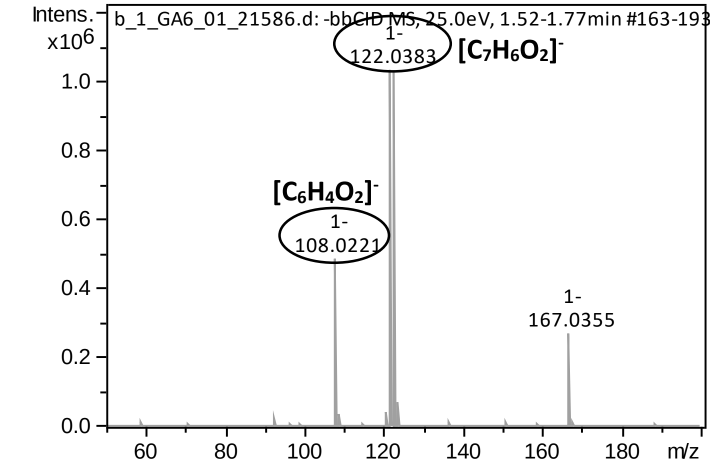

Fig S5d. Background subtracted MS/MS Spectra from 1.5 to 1.8 min

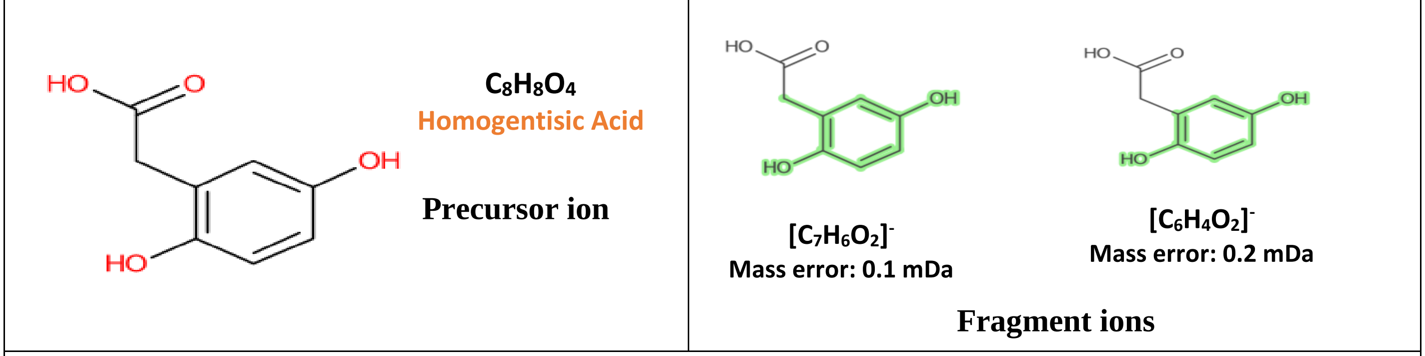

Fig S5e. Structures of precursor and fragment ions of Homogentisic Acid

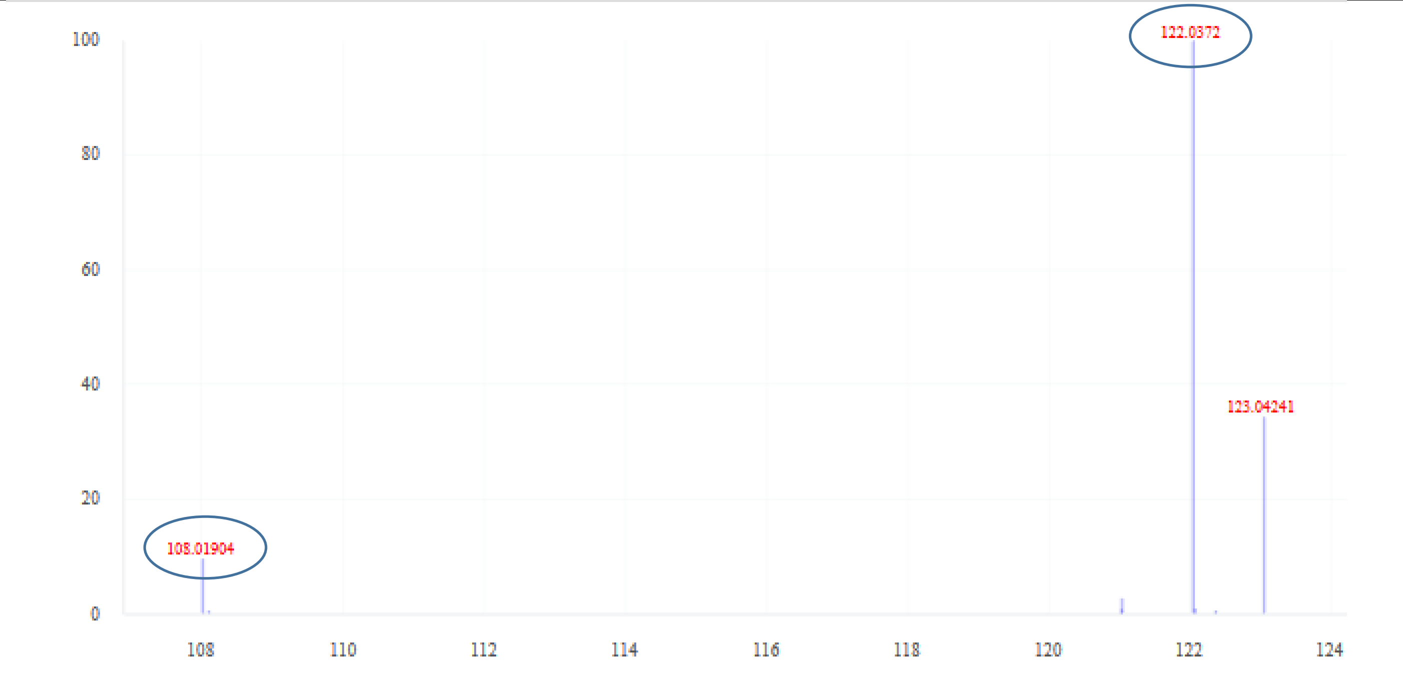

Fig. S5f: Fiehn Lab HILIC Library Record FiehnHILIC002750 (Homogentisic acid)

Figure S5. Identification data for the mass feature m/z 167.0350 _1.68 min (Homogentisic acid).

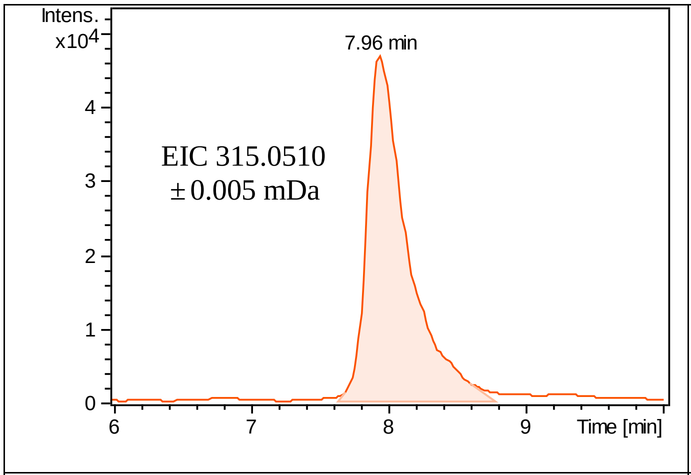

Fig S6a. EIC of m/z 315.0510 in a linden Honey

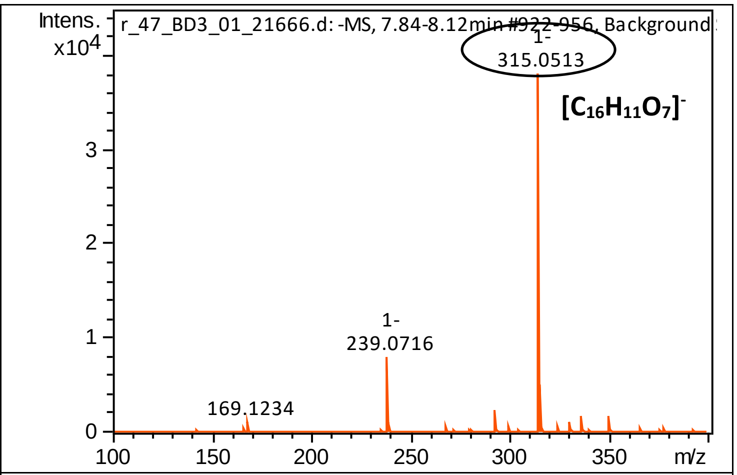

Fig S6b. Background subtracted MS Spectra from 7.8 to 8.1 min

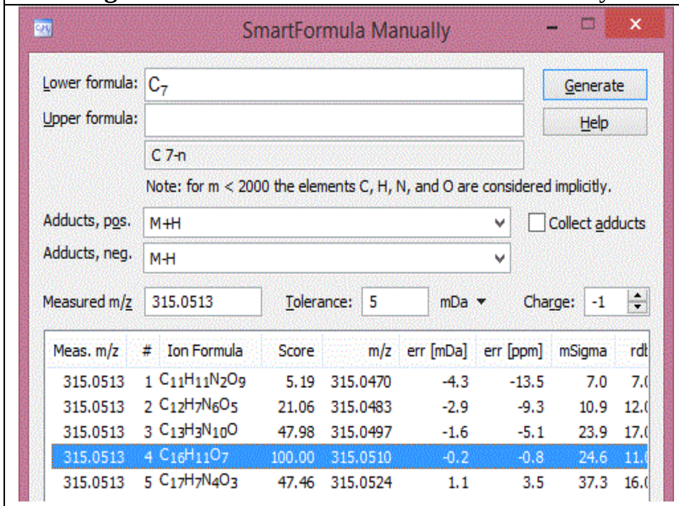

Fig S6c. Molecular Formula Annotation of m/z 315.0510

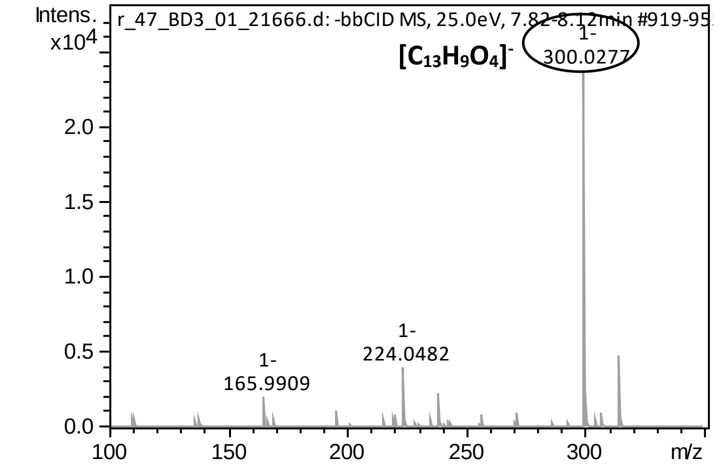

Fig S6d. Background subtracted MS/MS Spectra from 7.8 to 8.1 min

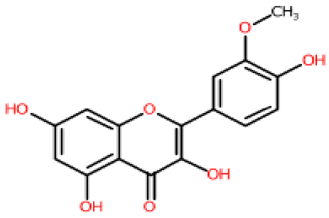

$C_{16}H_{12}O_7$

isorhamnetin

Precursor ion

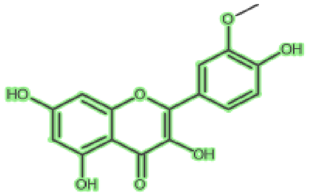

$[C_{15}H_8O_7]^-$

Mass error: 0.1 mDa

Fragment ions

Fig S6e. Structures of precursor and fragment ions of isorhamnetin

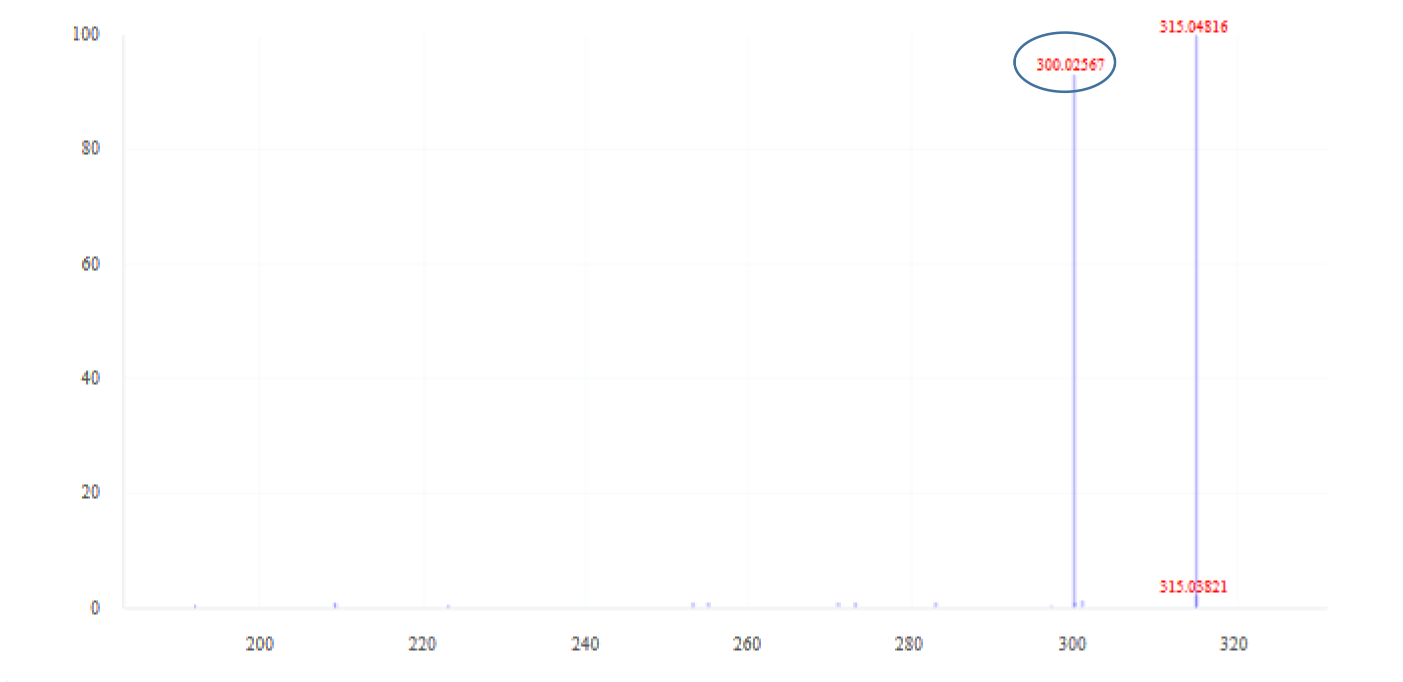

Fig. S6f: Fiehn Lab HILIC Library Record FiehnHILIC002780 (isorhamnetin)

Figure S6. Identification data for the mass feature m/z 315.0510_7.96 min (isorhamnetin).

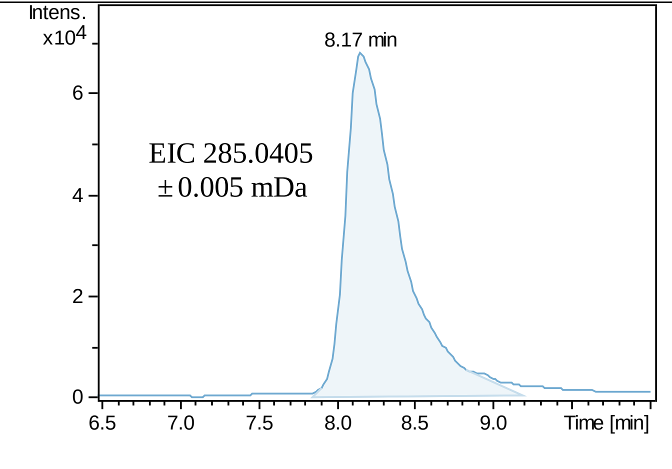

Fig S7a. EIC of m/z 285.0405 in a rape Honey

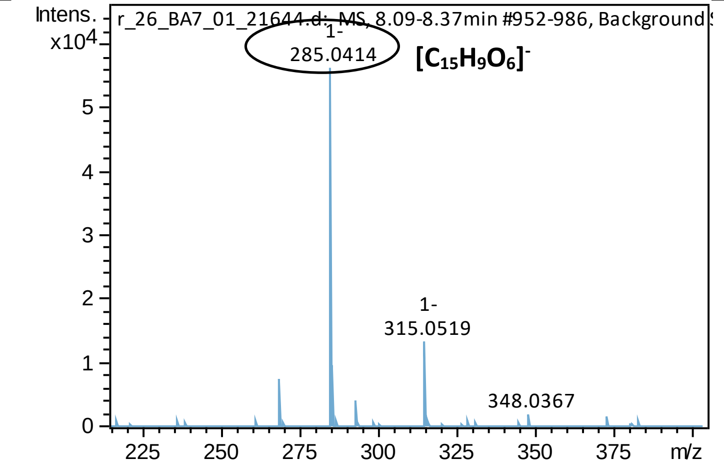

Fig S7b. Background subtracted MS Spectra from 8.1 to 8.3 min

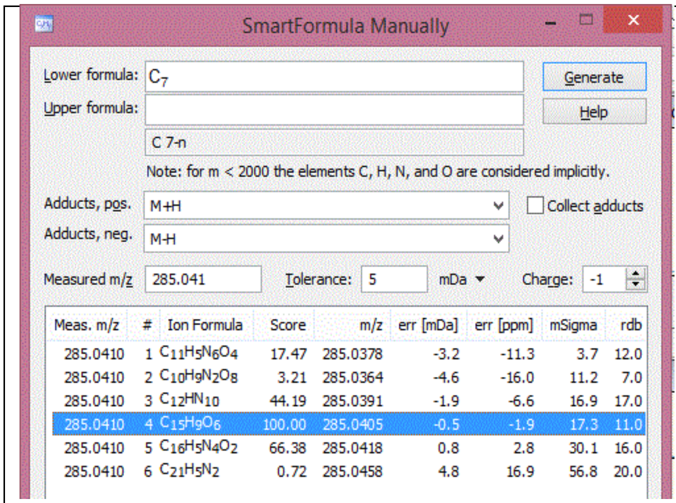

Fig S7c. Molecular Formula Annotation of m/z 285.0405

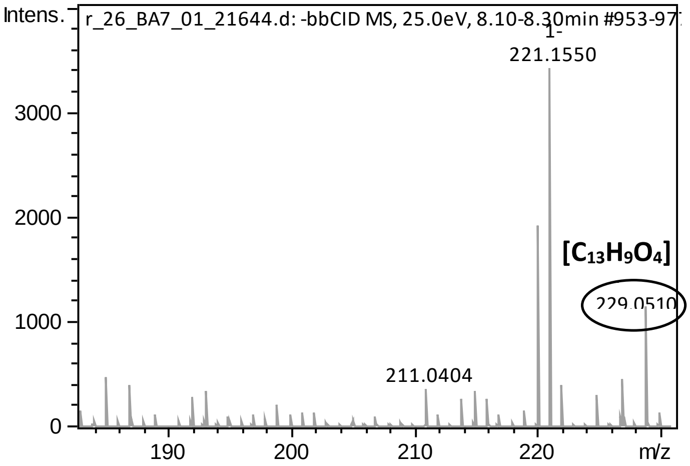

Fig S7d. Background subtracted MS/MS Spectra from 8.1 to 8.3 min

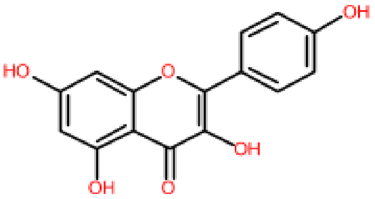

$C_{15}H_{10}O_5$

kaempferol

Precursor ion

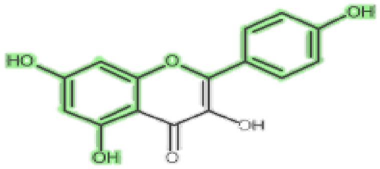

$[C_{13}H_9O_4]^-$

Mass error: 0.4 mDa

Fragment ion

Fig S7e. Structures of precursor and fragment ions of kaempferol

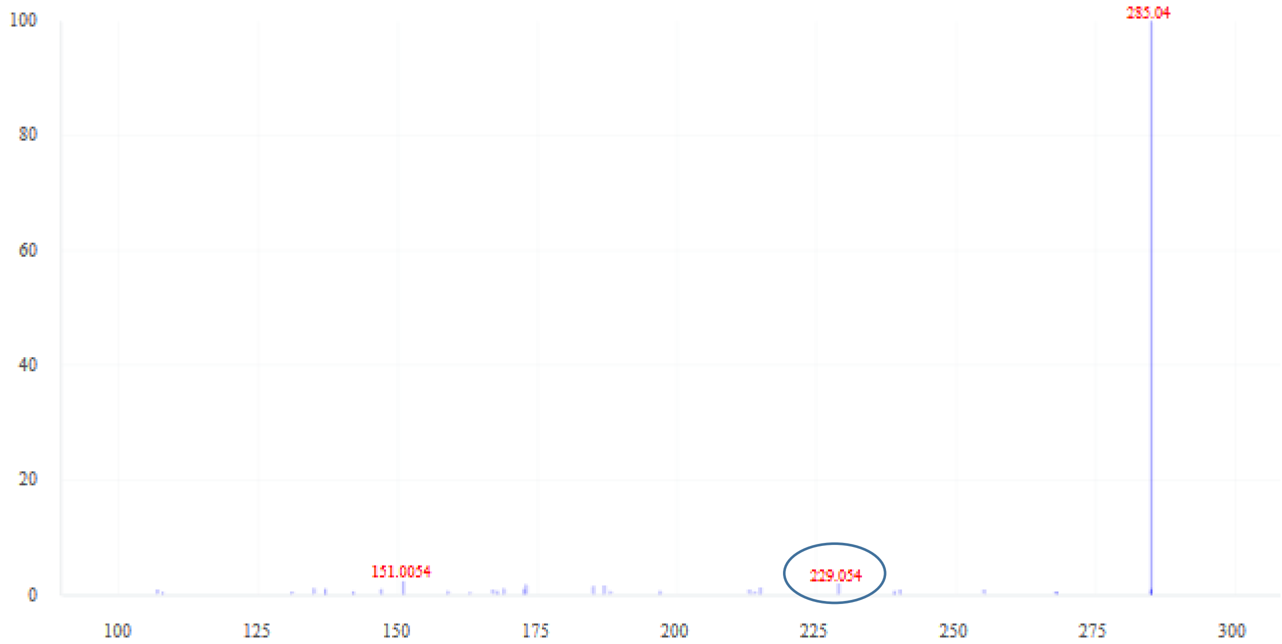

Fig. S7f: MassBank High Quality Mass Spectral Database Record BML00267 (Kaempferol)

Figure S7. Identification data for the mass feature m/z 285.0405_8.17 min (kaempferol).

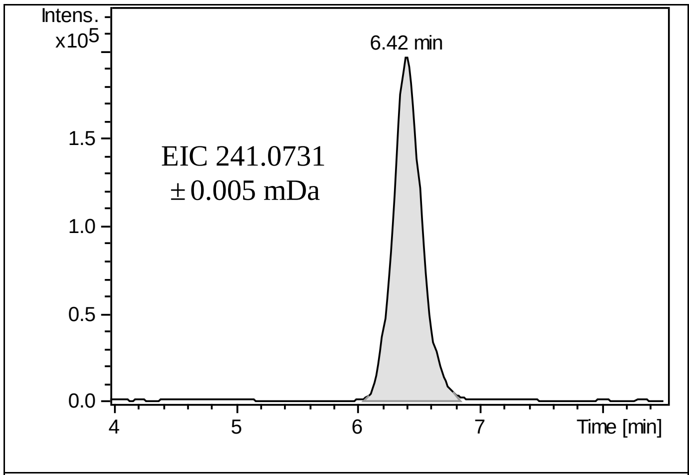

Fig S8a. EIC of m/z 241.0731 in heather Honey

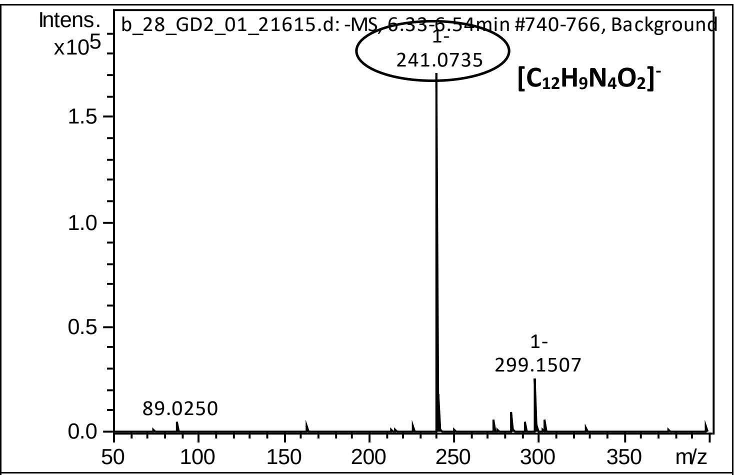

Fig S8b. Background subtracted MS Spectra from 6.3 to 6.5 min

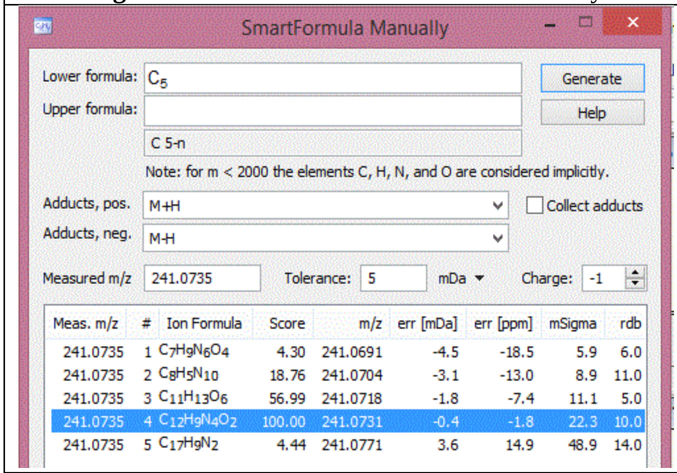

Meas. m/z	#	Ion Formula	Score	m/z	err [mDa]	err [ppm]	mSigma	rdb
241.0735	1	$C_7H_9N_6O_4$	4.30	241.0691	-4.5	-18.5	5.9	6.0
241.0735	2	$C_8H_5N_{10}$	18.76	241.0704	-3.1	-13.0	8.9	11.0
241.0735	3	$C_{11}H_{13}O_6$	56.99	241.0718	-1.8	-7.4	11.1	5.0
241.0735	4	$C_{12}H_9N_4O_2$	100.00	241.0731	-0.4	-1.8	22.3	10.0
241.0735	5	$C_{17}H_9N_2$	4.44	241.0771	3.6	14.9	48.9	14.0

Fig S8c. Molecular Formula Annotation of m/z 241.0731

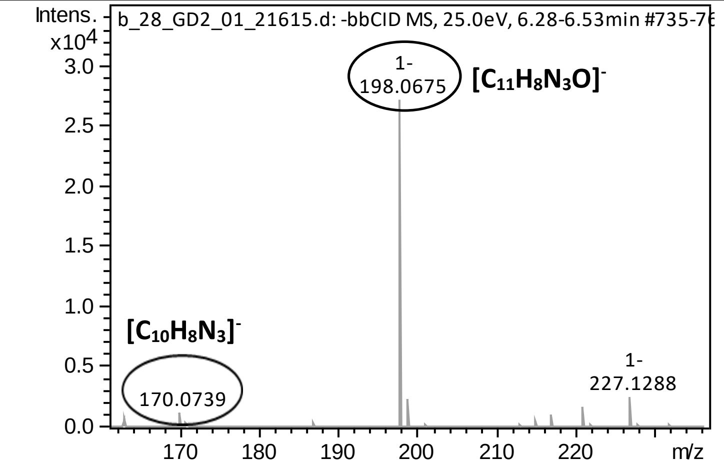

Fig S8d. Background subtracted MS/MS Spectra from 6.3 to 6.5 min

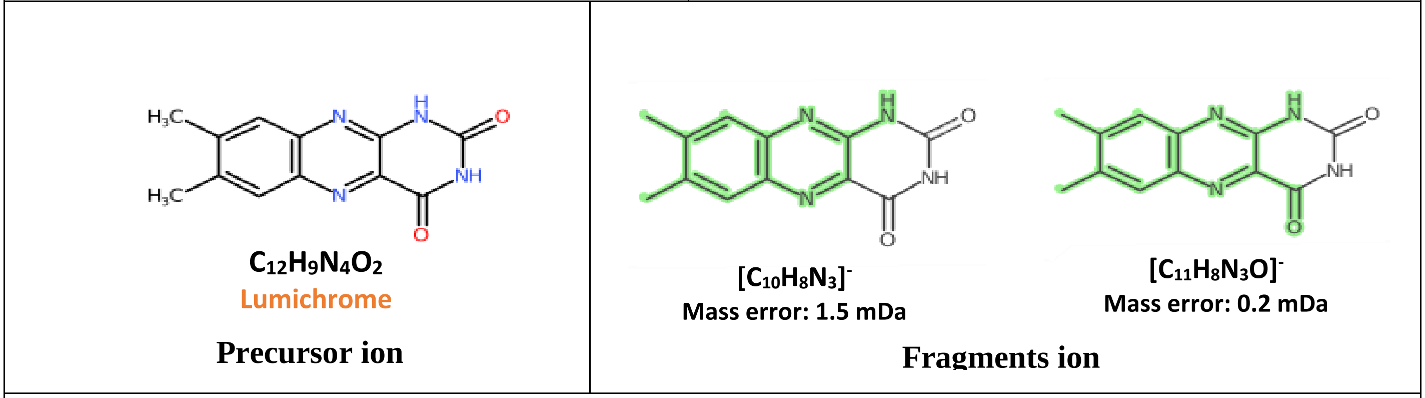

Fig S8e. Structures of precursor and fragment ions of Lumichrome

Figure S8. Identification data for the mass feature m/z 241.0731_6.42 min (Lumichrome).

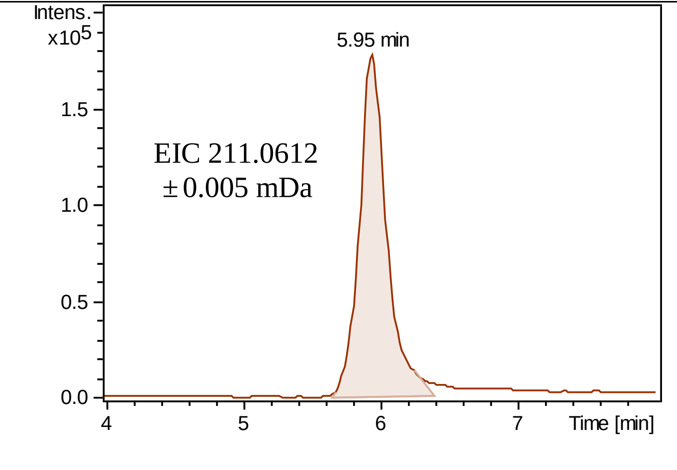

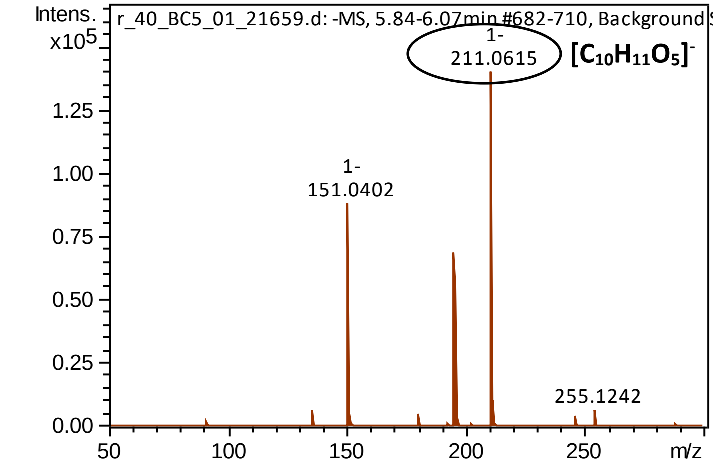

Fig S9a. EIC of m/z 211.0612 in rape Honey

Fig S9b. Background subtracted MS Spectra from 5.8 to 6.1 min

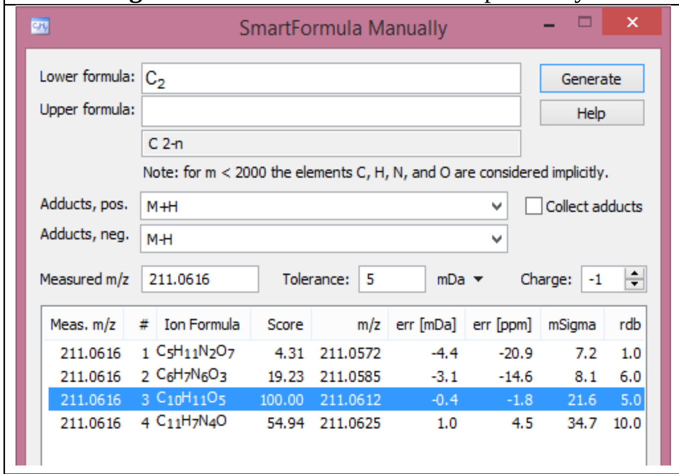

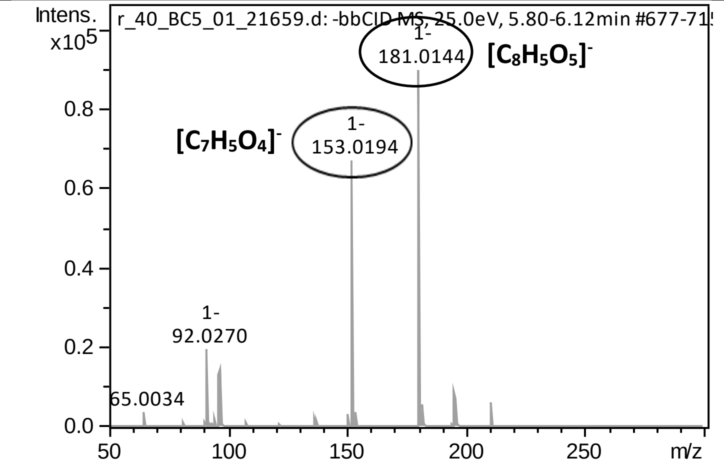

Fig S9c. Molecular Formula Annotation of m/z 211.0612

Fig S9d. Background subtracted MS/MS Spectra from 5.8 to 6.1 min

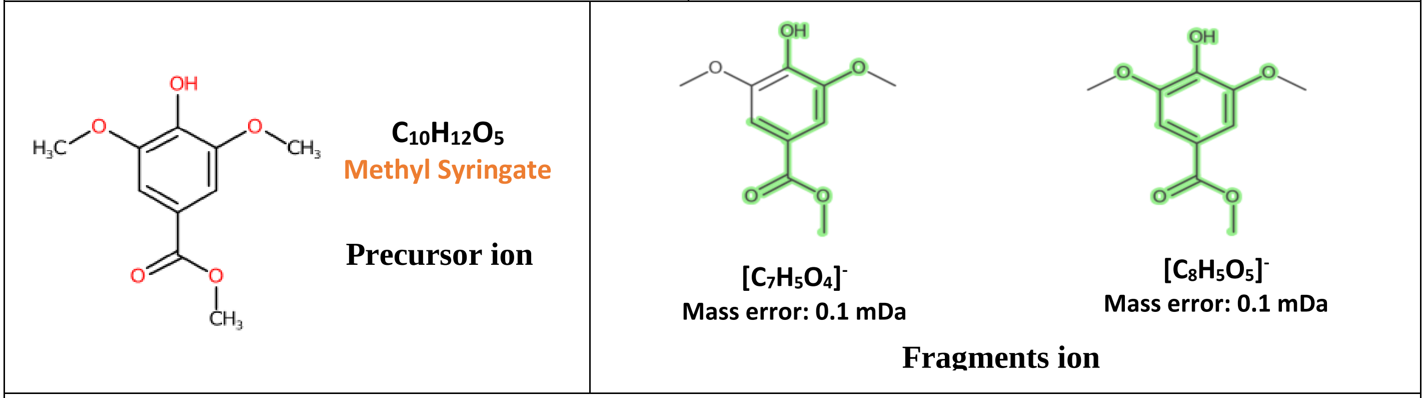

Fig S9e. Structures of precursor and fragment ions of Methyl Syringate

Figure S9. Identification data for the mass feature m/z 211.0612_5.96 min (Methyl Syringate).

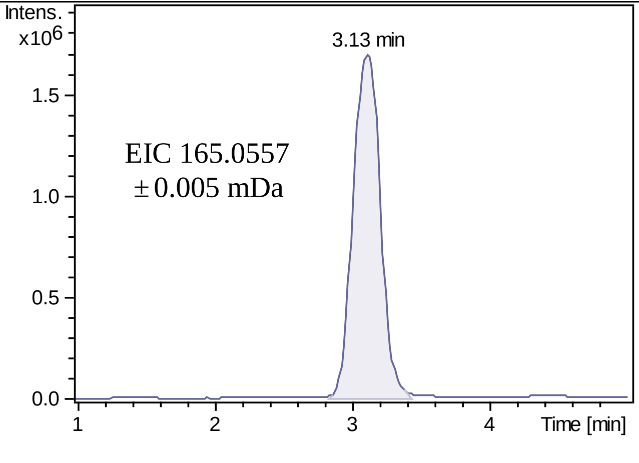

Fig S10a. EIC of m/z 165.0557 in a buckwheat Honey

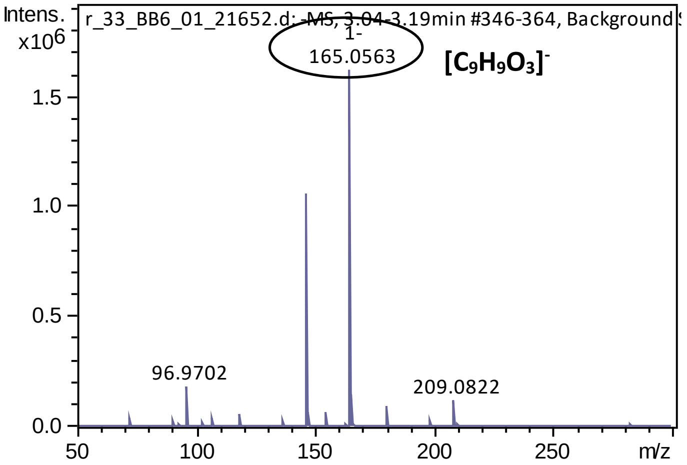

Fig S10b. Background subtracted MS Spectra from 3.0 to 3.2 min

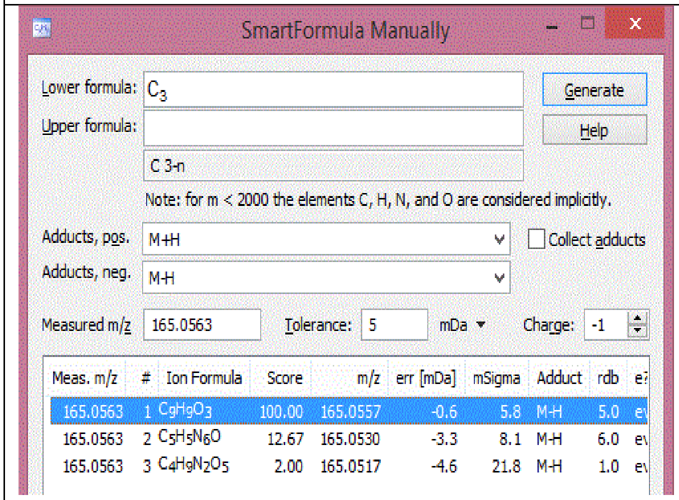

Fig S10c. Molecular Formula Annotation of m/z 165.0557

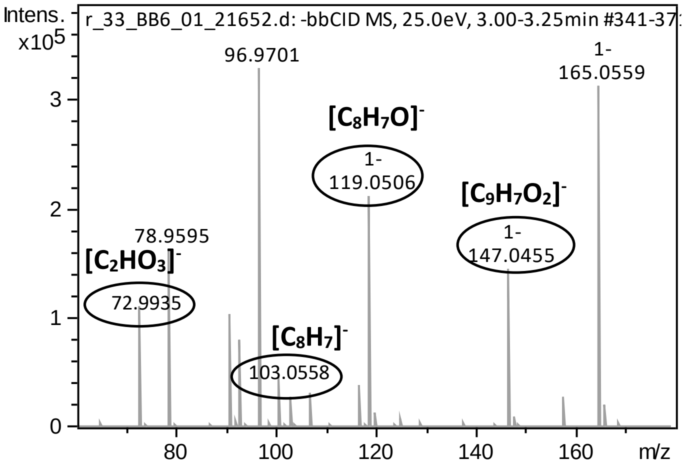

Fig S10d. Background subtracted MS/MS Spectra from 3.0 to 3.2 min

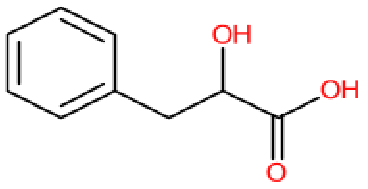

$C_9H_{10}O_3$

DL-β-Phenyllactic acid

Precursor ion

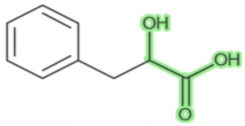

$[C_2HO_3]^-$

Mass error: 0.4 mDa

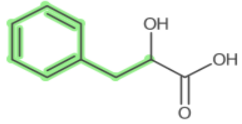

$[C_8H_7]^-$

Mass error: 0.4 mDa

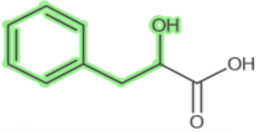

$[C_8H_7O]^-$

Mass error: 0.4 mDa

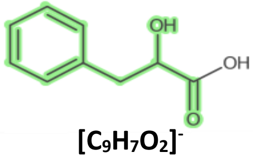

$[C_9H_7O_2]^-$

Mass error: 0.3 mDa

Fragment ions

Fig S10e. Structures of precursor and fragment ions of DL-β-Phenyllactic acid

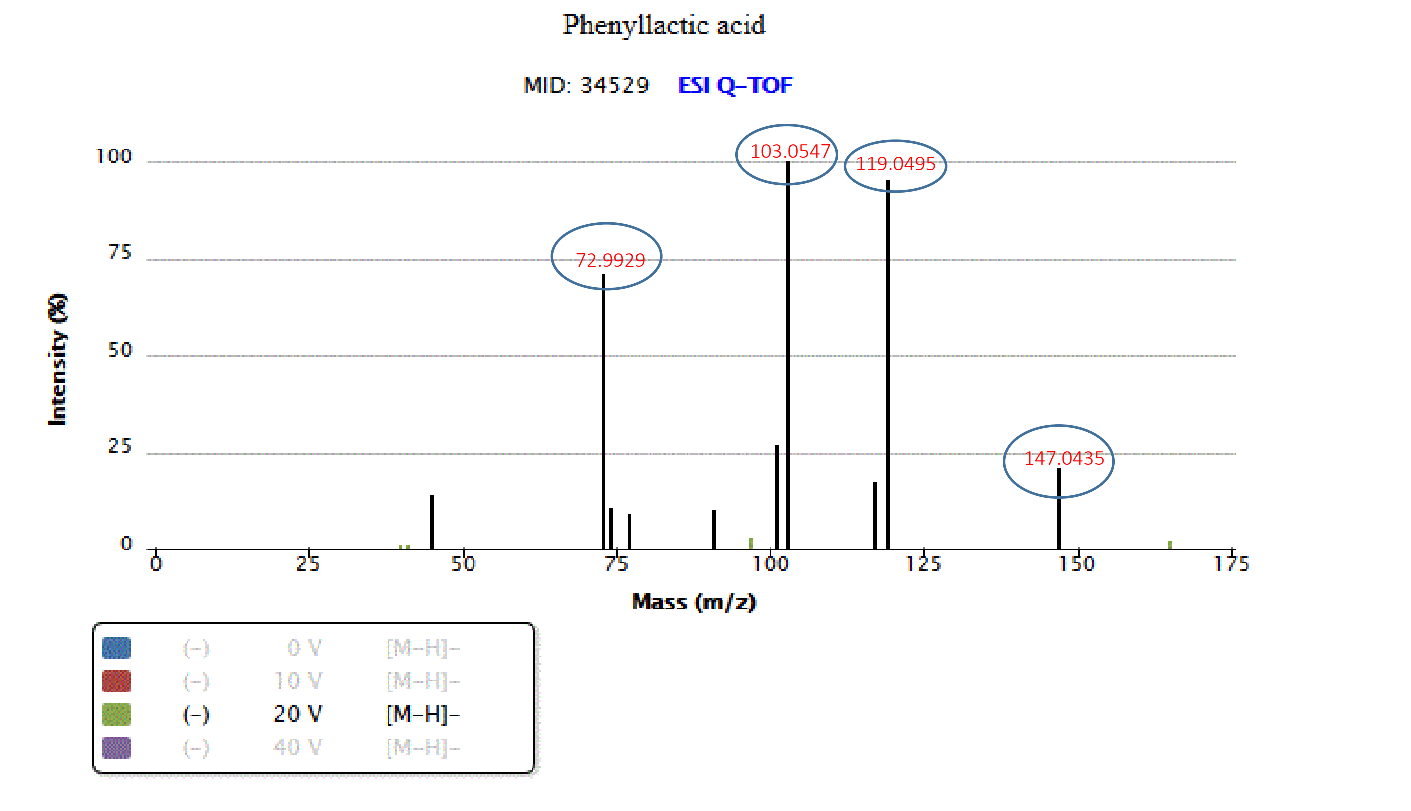

Fig. S10f: Metlin Library Record 34529 (DL-β-Phenyllactic acid)

Figure S10. Identification data for the mass feature m/z 165.0557_3.13 min (DL-β-Phenyllactic acid).

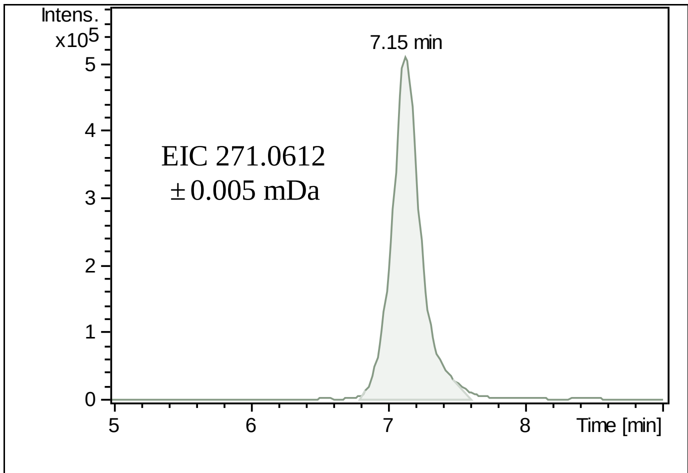

Fig S11a. EIC of m/z 271.0612 in a linden Honey

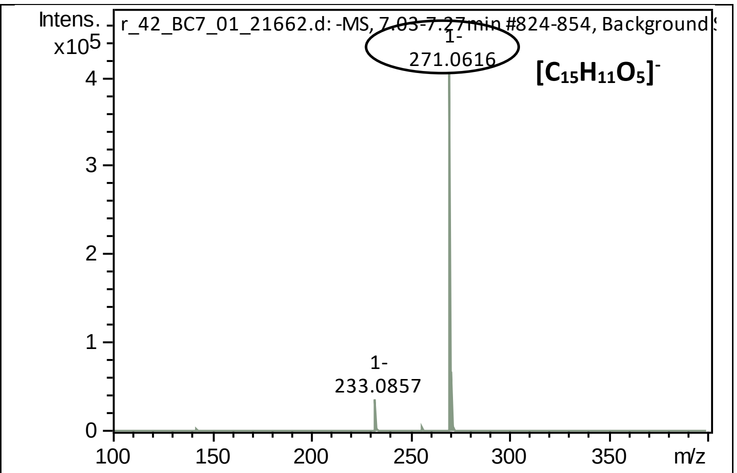

Fig S11b. Background subtracted MS Spectra from 7.0 to 7.3 min

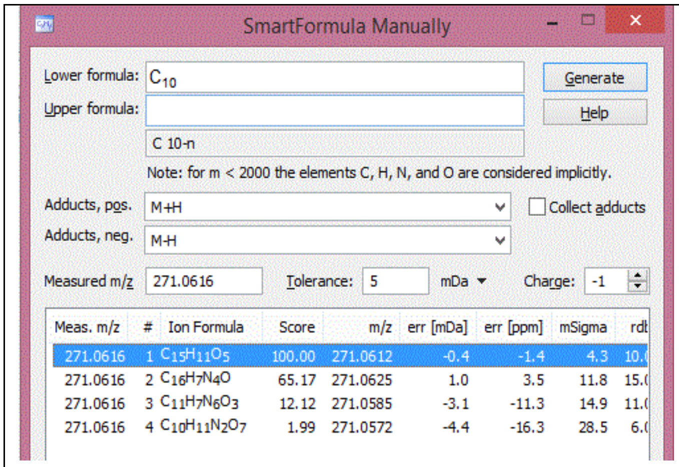

Fig S11c. Molecular Formula Annotation of m/z 271.0612

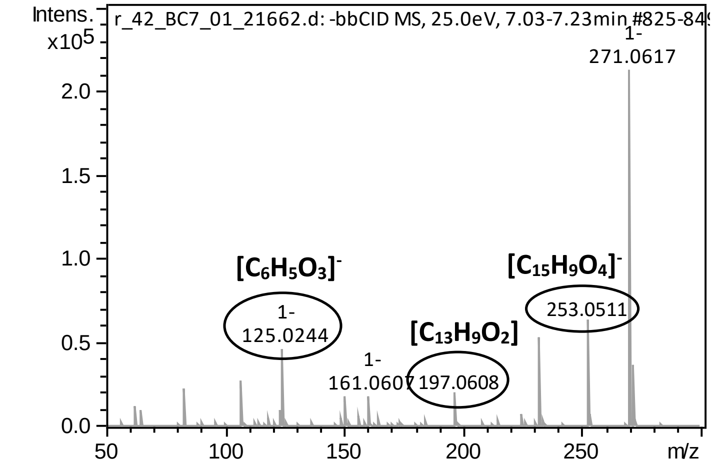

Fig S11d. Background subtracted MS/MS Spectra from 7.0 to 7.3 min

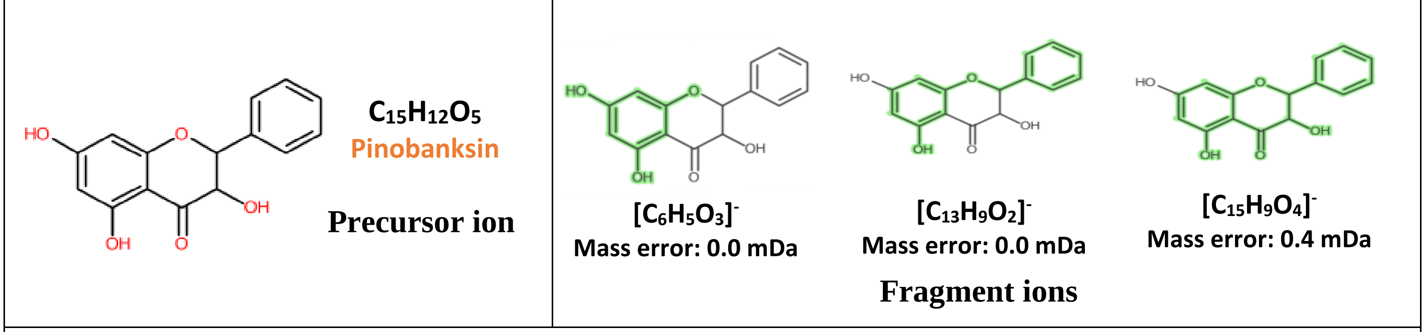

Fig S11e. Structures of precursor and fragment ions of Pinobanksin

Figure S11. Identification data for the mass feature m/z 271.0612_7.15 min (Pinobanksin).

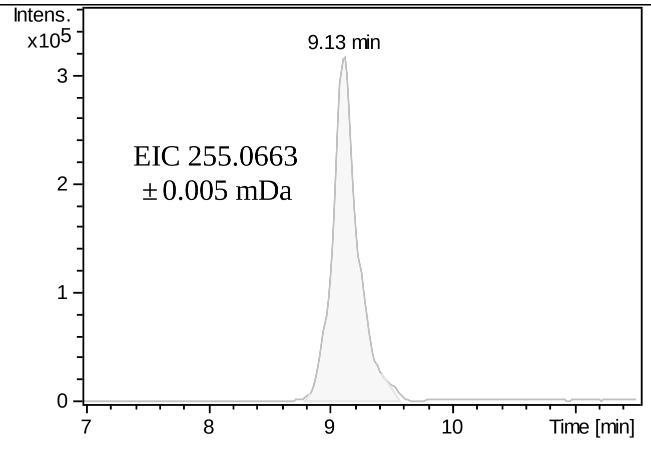

Fig S12a. EIC of m/z 255.0663 in acacia Honey

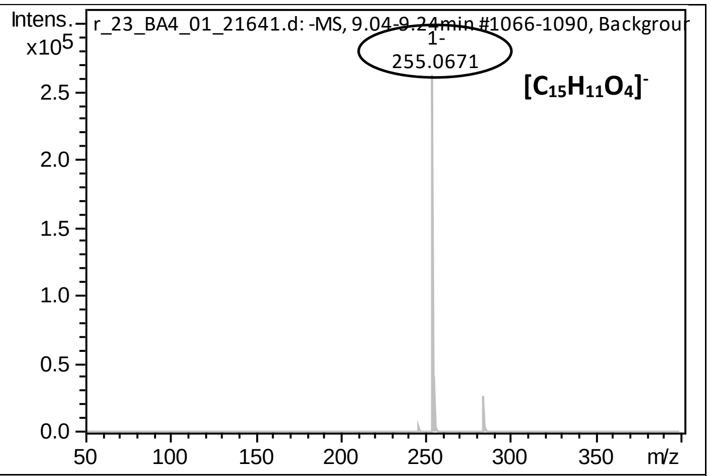

Fig S12b. Background subtracted MS Spectra from 9.0 to 9.3 min

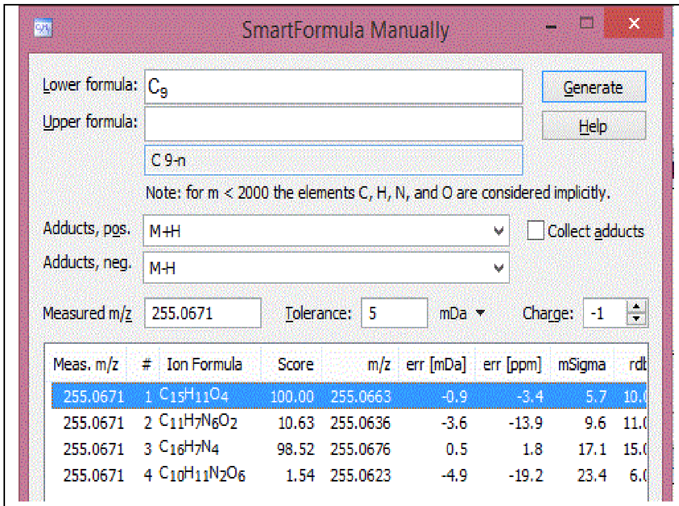

Fig S12c. Molecular Formula Annotation of m/z 255.0663

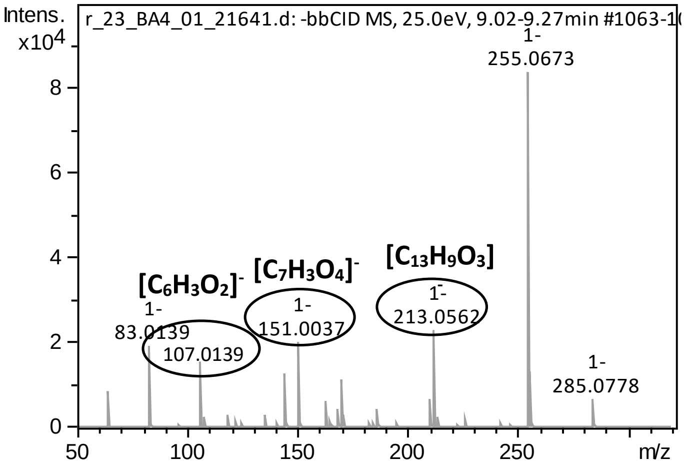

Fig S12d. Background subtracted MS/MS Spectra from 9.0 to 9.3 min

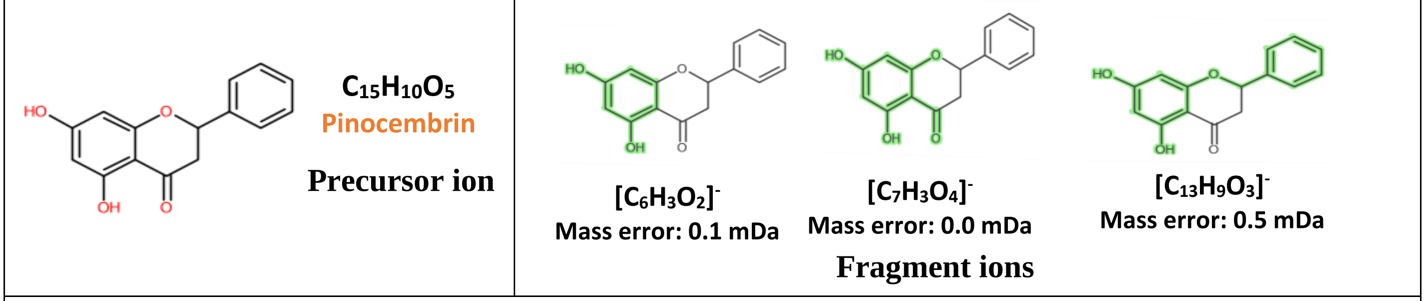

Fig S12e. Structures of precursor and fragment ions of Pinocembrin

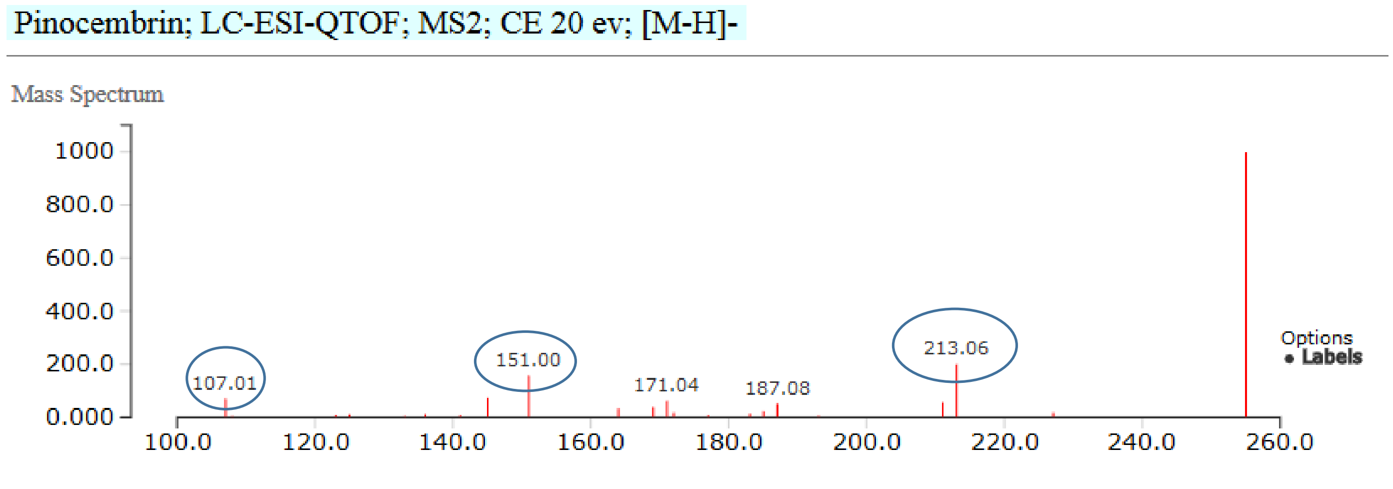

Fig. S12f: Massbank Library Record BML00897 (Pinocembrin)

Figure S12. Identification data for the mass feature m/z 255.0663_9.13 min (Pinocembrin).

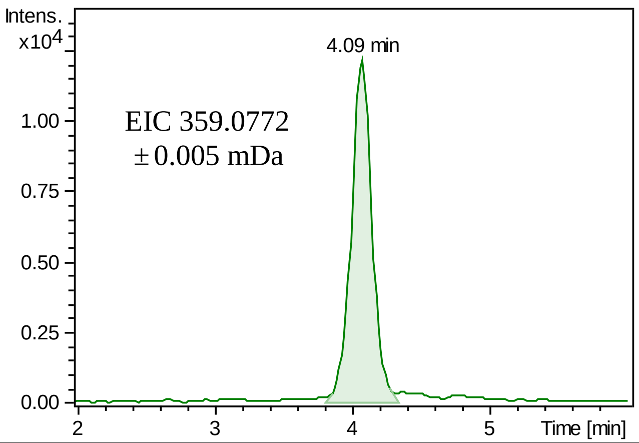

Fig S13a. EIC of m/z 359.0772 in thyme Honey

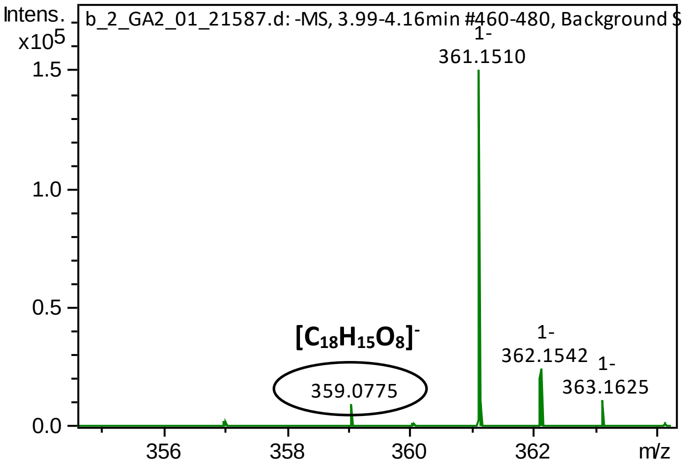

Fig S13b. Background subtracted MS Spectra from 4.0 to 4.2 min

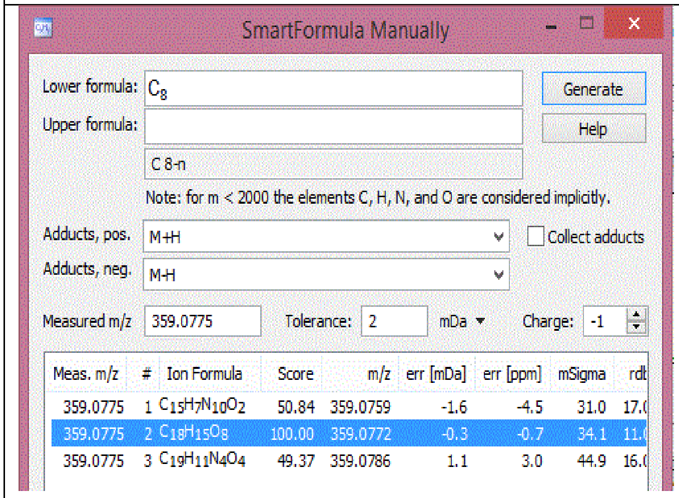

Fig S13c. Molecular Formula Annotation of m/z 359.0772

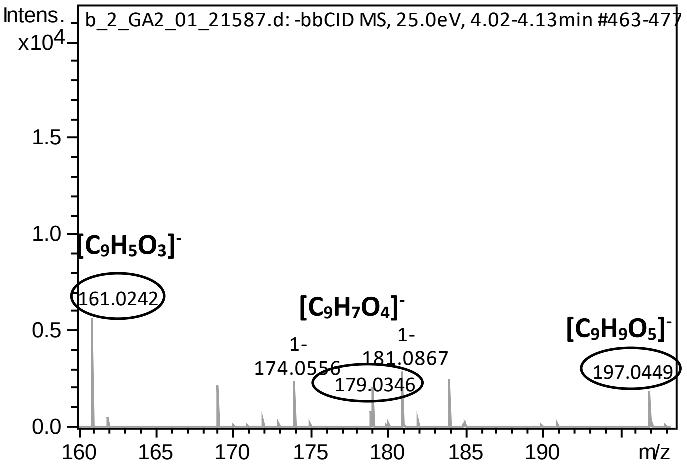

Fig S13d. Background subtracted MS/MS Spectra from 4.0 to 4.2 min

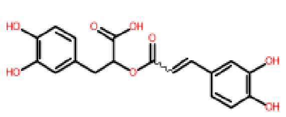

$C_{18}H_{16}O_8$

Rosmarinic Acid

Precursor ion

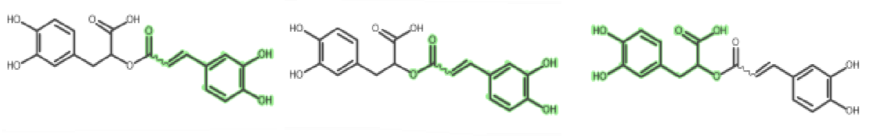

$[C_9H_5O_3]^-$
Mass error: 0.3 mDa

$[C_9H_7O_4]^-$
Mass error: 0.4 mDa

$[C_9H_9O_5]^-$
Mass error: 0.7 mDa

Fragment ions

Fig S13e. Structures of precursor and fragment ions of Rosmarinic Acid

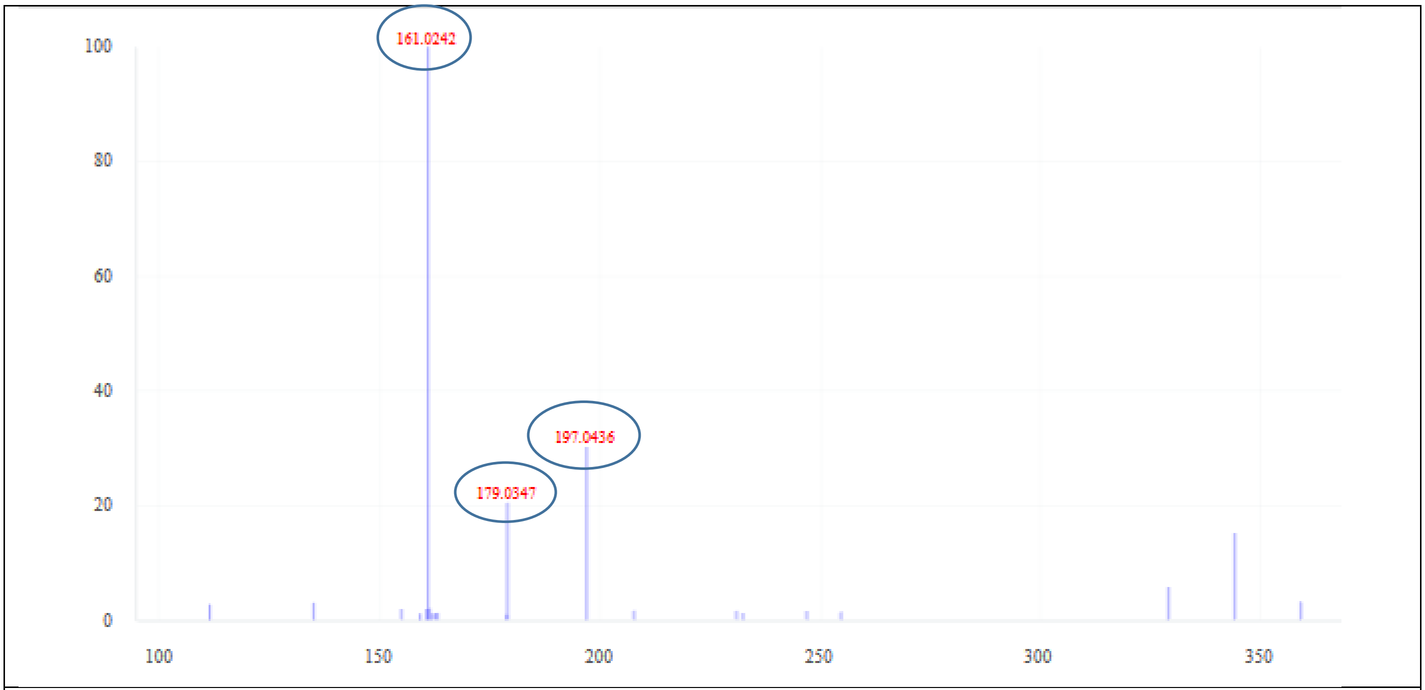

Fig. S13f: Vaniya/Fiehn Natural Products Library Record VF-NPL-QTOF009534 (Rosmarinic Acid)

Figure S13. Identification data for the mass feature m/z 359.0772_4.09 min (Rosmarinic Acid).

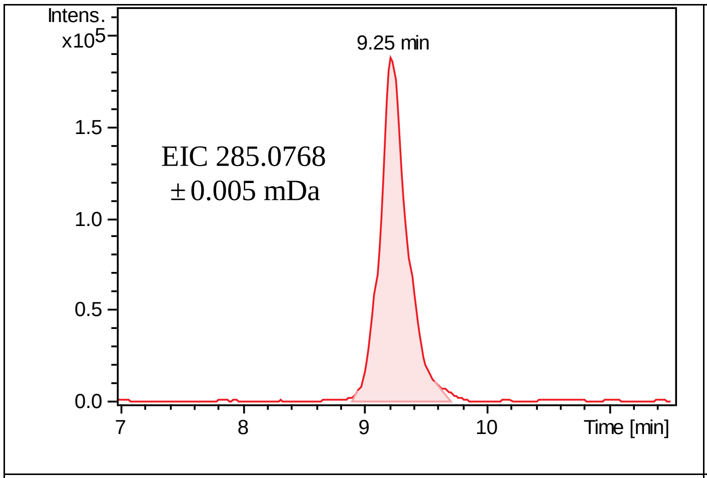

Fig S14a. EIC of m/z 285.0768 in heather Honey

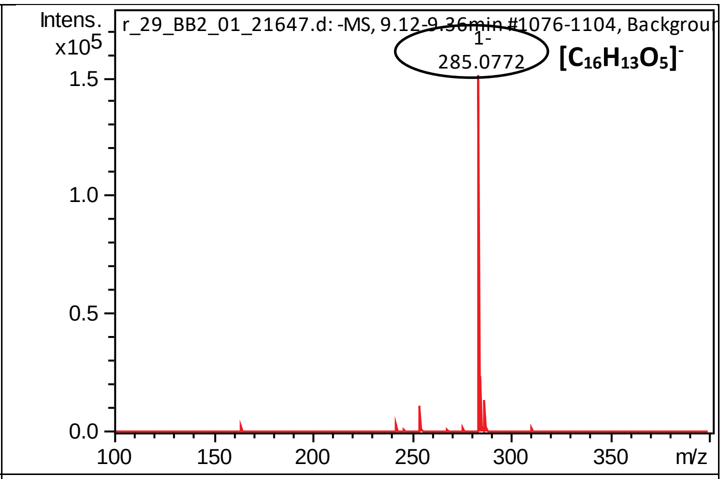

Fig S14b. Background subtracted MS Spectra in 9.1-9.4 min

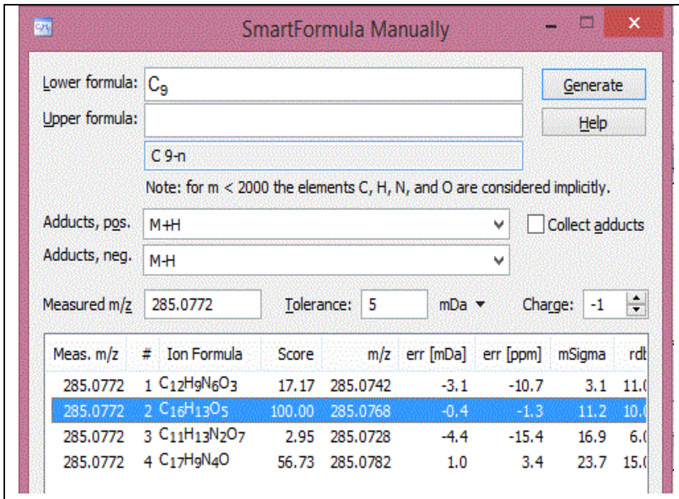

Fig S14c. Molecular Formula Annotation of m/z 285.0768

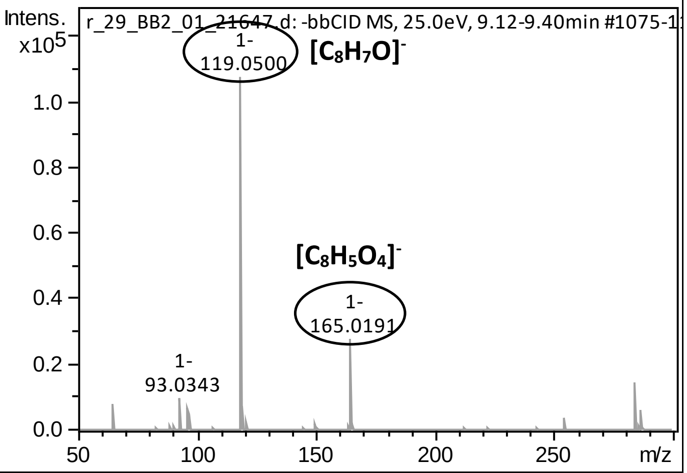

Fig S14d. Background subtracted MS/MS Spectra in 9.1-9.4 min

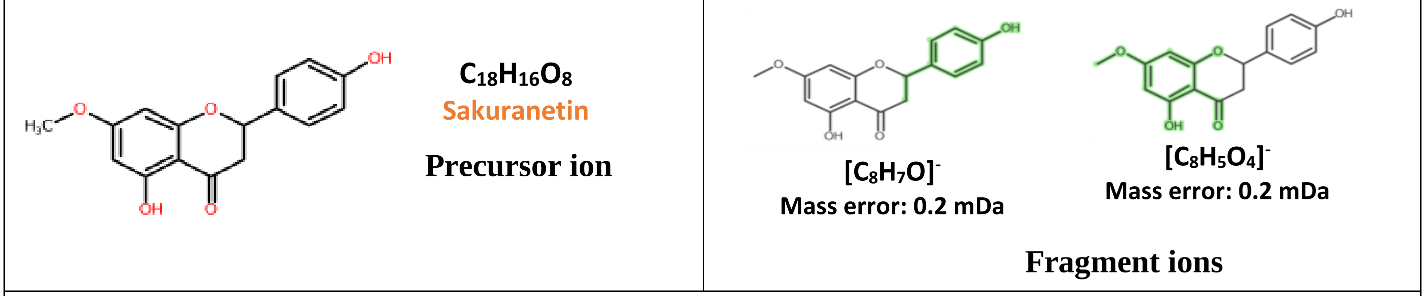

Fig S14e. Structures of precursor and fragment ions of Sakuranetin

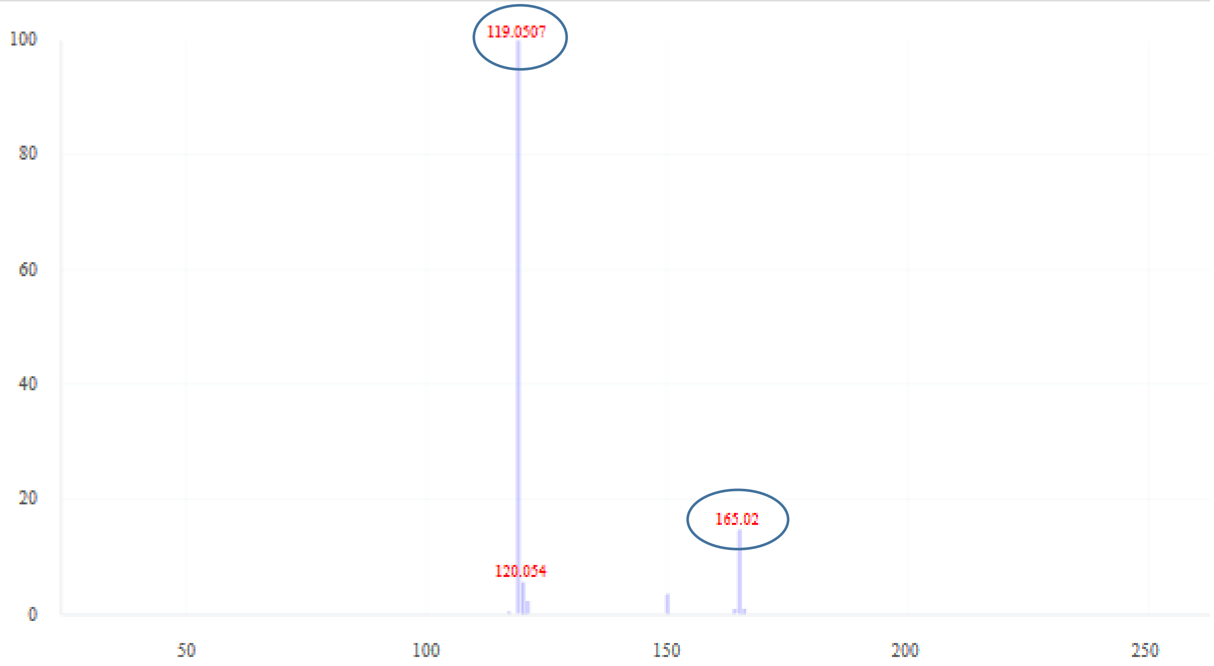

Fig. S14f: Bruker Sumner MetaboBASE Plant Library Record MetaboBASE0561 (Sakuranetin)

Figure S14. Identification data for the mass feature m/z 285.0768_9.25 min (Sakuranetin).

Table S4. Number of samples of each botanical origin which have been identified each compound

Compound	acacia (#/6)	arbutus (#/2)	blossom (#/2)	buckwheat (#/8)	chestnut (#/2)	fir (#/2)	heather (#/10)	linden (#/9)	rape (#/7)	thyme (#/3)
2-trans,4-trans-abscisic acid	6	2	2	8	2	2	10	9	7	3
2-cis,4-trans-abscisic acid	6	2	2	8	2	2	10	9	7	3
Acacetin	6	1	0	8	0	2	9	7	6	3
Chrysin	6	2	2	8	2	2	10	9	7	3
Dehydrovomifoliol	3	0	2	8	1	2	10	4	5	3
Galangin	6	2	2	8	2	2	10	9	7	3
Homogentisic Acid	0	2	0	4	0	0	2	6	0	0
isorhamnetin	6	2	2	8	2	2	9	9	7	3
Kaempferol	5	1	2	8	1	2	7	8	7	3
Lumichrome	3	1	2	7	1	1	9	8	6	1
Methyl Syringate	5	0	2	7	2	1	6	9	7	3
Phenyllactic Acid	5	0	2	8	2	1	9	9	7	3
Pinobanksin	6	2	2	8	2	2	9	9	7	3
Pinocembrin	6	2	2	8	2	2	9	9	7	3
Rosmarinic Acid	0	0	0	0	0	0	0	0	0	1
Sakuranetin	6	2	2	8	2	2	9	9	7	3

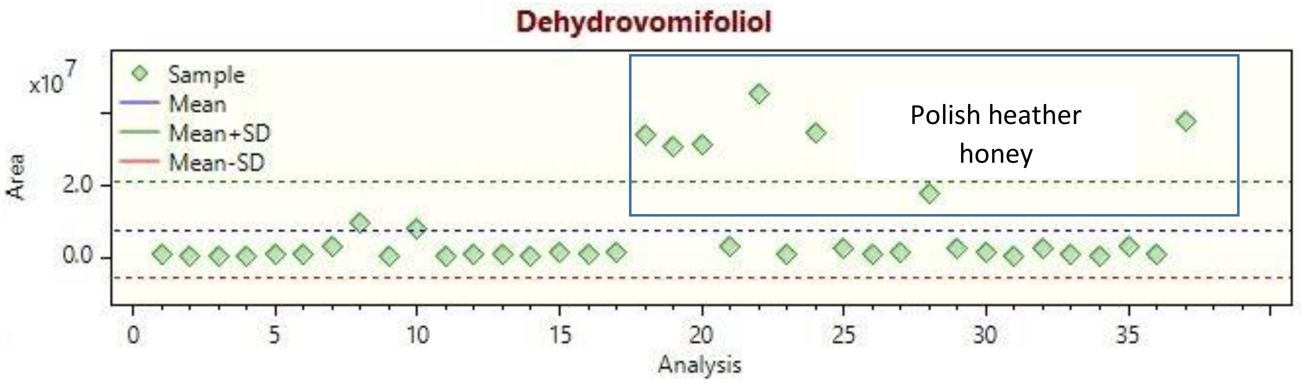

Figure S15. Batch statistics graph showing the mean area and the standard deviation for Dehydrovomifoliol

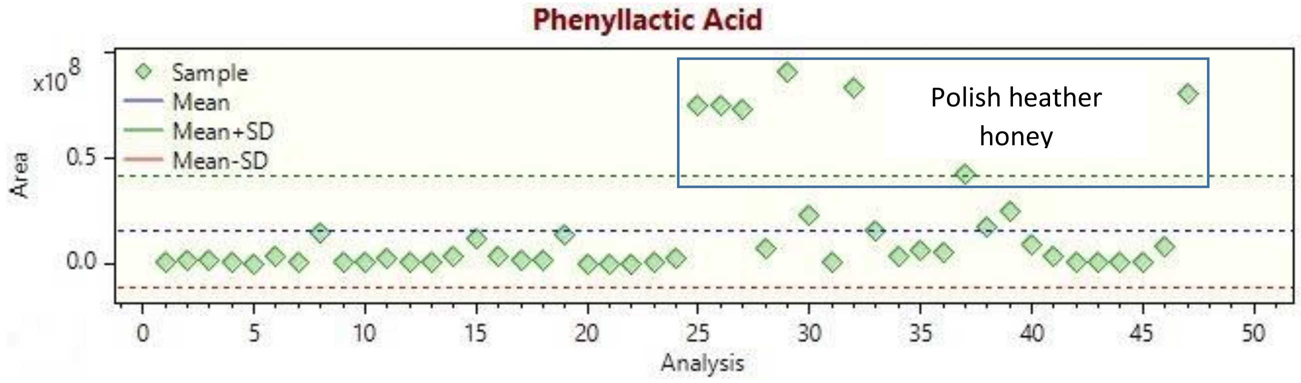

Figure S16. Batch statistics graph showing the mean area and the standard deviation for Phenyllactic acid

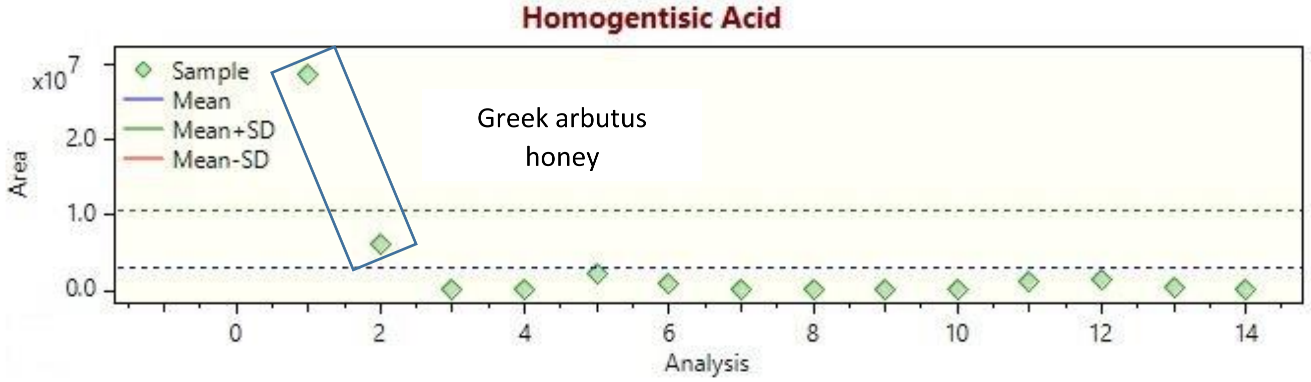

Figure S17. Batch statistics graph showing the mean area and the standard deviation for Homogentisic acid

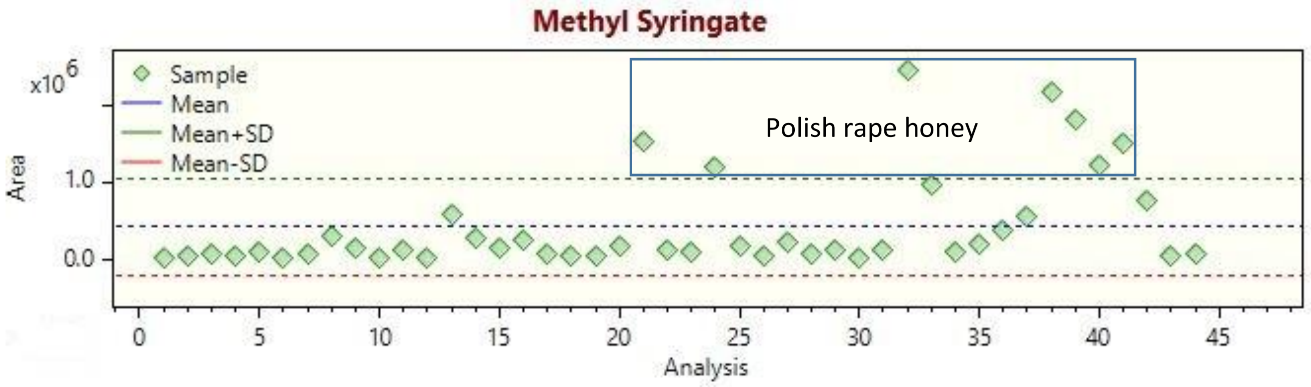

Figure S18. Batch statistics graph showing the mean area and the standard deviation for Methyl syringate

Section S4: Honey Samples

Table S5. Honey samples characterization

Botanical origin	Production type	Geographical origin
acacia	conventional	Poland
acacia	organic	Poland
acacia	organic	Poland
acacia	conventional	Poland
acacia	conventional	Poland
acacia	conventional	Poland
arbutus	organic	Greece
arbutus	organic	Greece
blossom	organic	Greece
blossom	conventional	Greece
buckwheat	conventional	Poland
buckwheat	organic	Poland
buckwheat	organic	Poland
buckwheat	organic	Poland
buckwheat	conventional	Poland
buckwheat	conventional	Poland
buckwheat	conventional	Poland
buckwheat	conventional	Poland
chestnut	organic	Greece
chestnut	conventional	Greece
fir	organic	Greece
fir	organic	Greece
heather	conventional	Poland
heather	conventional	Poland
heather	conventional	Poland
heather	conventional	Poland
heather	conventional	Poland
heather	conventional	Poland
heather	conventional	Poland
heather	organic	Greece
heather	organic	Greece
heather	conventional	Greece
linden	conventional	Poland
linden	organic	Poland
linden	organic	Poland
linden	organic	Poland
linden	conventional	Poland

linden	conventional	Poland
linden	conventional	Poland
linden	conventional	Poland
linden	conventional	Poland
rape	organic	Poland
rape	conventional	Poland
rape	conventional	Poland
rape	conventional	Poland
rape	conventional	Poland
rape	conventional	Poland
rape	conventional	Poland
thyme	organic	Greece
thyme	organic	Greece
thyme	organic	Greece

Section S5: LC elution program

Table S6. LC gradient elution and flow rate program

Time (min)	Flow rate (mL/min)	%A	%B
0	0.2	99	1
1.0	0.2	99	1
3.0	0.2	61	39
14.0	0.4	0.1	99.9
16.0	0.48	0.1	99.9
16.1	0.48	99	1
19.0	0.48	99	1
19.1	0.2	99	1
20.0	0.2	99	1

Section S6: Validation procedure

Accuracy is one of the critical parameters to be assessed for method validation, and it is estimated through trueness and precision. For the assessment of trueness, the recovery of known amounts of intentionally added analytes to the blank matrix has been calculated. Thus, the samples were fortified at a middle concentration level of 0.4 mg/Kg, and the extraction procedure was followed. The recovery of each analyte was calculated by the following formula:

R%= (peak area of spiked sample / peak area of matrix-matched standard) x 100

Regarding precision, six replicates at the concentration of 0.4 mg/Kg were analyzed during the same laboratory day for the assessment of method repeatability (n=6), while for the evaluation of intermediate precision, the same experiment was conducted during two more analysis days (n=18). The RSD% of repeatability and intermediate precision were calculated.

The measurement of matrix effect (ME) is an important issue as the selectivity of a proposed method is investigated. The ME was determined by the comparison of the peak area of each analyte in a matrix-match standard at the above-mentioned concentration level subtracting the area of the analyte in the blank sample with that in a standard prepared in solvent at the same concentration according to the formula:

Matrix Effect% = [1- (Peak area in the matrix-matched standard - Peak area in the blank sample/ Peak area in the standard)] × 100.

The linearity was determined by the least-squares method using the matrix-matched calibration curve. Finally, LODs and LOQs were calculated from the matrix-matched calibration curve using the standard deviation of the response (Sy) and the slope of the calibration curve (S) according to the formula: LOD = 3.3*(Sy/S) and LOQ = 10*(Sy/S).

Section S7: Suspect database

Table S7. Suspect list of bioactive compounds encountered in honey

ID	Compound name	Molecular Formula	Predicted tR (min)	Monoisotopic Mass	$[M]^-$	$[M-H]^-$	$[M-H_2O-H]^-$	$[M+Cl]^-$	$[M+HCOOH-H]^-$	$[M+CH_3COOH-H]^-$	Qual. 1	Qual. 2	Qual. 3	Qual. 4	Qual. 5	Reference
1	Genistein	$C_{15}H_{10}O_5$	7.86	270.0528	270.0534	269.0455	251.0350	305.0222	315.051	329.0667	269.0456	225.0571	181.0661	133.0270	201.0563	3
2	Pinocembrin	$C_{15}H_{12}O_4$	8.14	256.0736	256.0741	255.0663	237.0557	291.043	301.0718	315.0874	145.0671	255.0666	213.0552	151.0035	171.0441	3
3	Tricetin	$C_{15}H_{10}O_7$	7.61	302.0427	302.0432	301.0354	283.0248	337.0121	347.0409	361.0565	301.0407	302.0430	303.0484	149.0275		3
4	Chrysin	$C_{15}H_{10}O_4$	8.15	254.0579	254.0585	253.0506	235.0401	289.0273	299.0561	313.0718	143.0485	145.0277	253.0501	209.0617	107.0151	3
5	Quercetin 3-Methyl Ether	$C_{16}H_{12}O_7$	7.07	316.0583	316.0589	315.0510	297.0405	351.0277	361.0565	375.0722	271.0269	300.0280	255.0342			3
6	Quercetin 3,4-Diglycoside	$C_{27}H_{30}O_{17}$	5.60	626.1483	626.1488	625.1410	607.1305	661.1177	671.1465	685.1622	463.0887	301.0354	300.0293	625.1392	179.0007	3
7	Quercetin-O-Rhamnoside	$C_{21}H_{20}O_{11}$	7.03	448.1006	448.1011	447.0933	429.0827	483.0700	493.0988	507.1144	447.0979	301.0380	255.0305	300.0291	284.0323	3
8	Kaempferol	$C_{15}H_{10}O_6$	7.26	286.0477	286.0483	285.0405	267.0299	321.0171	331.0459	345.0616	285.0399	159.0378	117.0346	286.0442	151.0034	3
9	Kaempferol 3-Ome	$C_{16}H_{12}O_6$	7.48	300.0634	300.0639	299.0561	281.0455	335.0328	345.0616	359.0772						3
10	Kaempferol-7-O-Rhamnoside	$C_{21}H_{20}O_{10}$	7.51	432.1056	432.1062	431.0984	413.0878	467.0750	477.1038	491.1195	285.0399	284.0326	431.0991	151.0041		3
11	Kaempferol-3-O-Glycosyl	$C_{21}H_{20}O_{11}$	5.93	448.1006	448.1011	447.0933	429.0827	483.0700	493.0988	507.1144	447.0979	301.038	255.0305	300.0291	284.0323	3
12	Kaempferol-3-Rhamnoside	$C_{21}H_{20}O_{10}$	7.10	431.0978	431.0984	430.0905	412.0800	466.0672	476.0960	490.1117						64
13	Kaempferol-7-O-Glycosyl	$C_{21}H_{20}O_{11}$	6.25	448.1006	448.1011	447.0933	429.0827	483.0700	493.0988	507.1144	447.0931	285.0443	284.0285	150.9993	286.0430	3
14	Galangin	$C_{15}H_{10}O_5$	7.48	270.0528	270.0534	269.0455	251.0350	305.0222	315.0510	329.0667	269.0466	227.0356	270.0498	271.0509	211.0380	65
15	Pinobanksin	$C_{15}H_{12}O_5$	7.35	272.0685	272.0690	271.0612	253.0506	307.0379	317.0667	331.0823						65
16	Myricetin 3-Ome	$C_{16}H_{12}O_8$	6.91	332.0532	332.0538	331.0459	313.0354	367.0226	377.0514	391.0671						3
17	Isorhamnetin	$C_{16}H_{12}O_7$	7.63	316.0583	316.0589	315.0510	297.0405	351.0277	361.0565	375.0722	315.0504	300.0272	151.0038	107.0134	108.0212	66
18	Naringenin	$C_{15}H_{12}O_5$	7.96	272.0685	272.0690	271.0612	253.0506	307.0379	317.0667	331.0823	151.0038	119.0501	107.0135	271.0606	177.0196	66
19	4-Dimethylaminobenzoic Acid	$C_9H_{11}NO_2$	3.21	165.0790	165.0795	164.0717	146.0611	200.0484	210.0772	224.0928						3
20	Chlorogenic Acid	$C_{16}H_{18}O_9$	3.70	354.0951	354.0956	353.0878	335.0772	389.0645	399.0933	413.1089	191.0575	353.0866	179.0378	161.0270	87.0068	3
21	Homogentisic Acid	$C_8H_8O_4$	3.29	168.0423	168.0428	167.0350	149.0244	203.0117	213.0405	227.0561	123.0441	122.0361	108.0203	167.0342	124.0472	67
22	Methyl Syringate	$C_{10}H_{12}O_5$	5.77	212.0685	212.0690	211.0612	193.0506	247.0379	257.0667	271.0823						69
23	Acacetin	$C_{16}H_{12}O_5$	7.99	284.0685	284.0690	283.0612	265.0506	319.0379	329.0667	343.0823	268.0362	283.0609	269.0339	284.0623	240.0442	70
24	2-trans,4-trans-abscisic acid	$C_{15}H_{20}O_4$	4.44	264.1362	264.1367	263.1289	245.1183	299.1056	309.1344	323.1500	153.0914	219.1382	204.1150	201.1269	203.1059	71
25	2-cis,4-trans-abscisic acid	$C_{15}H_{20}O_4$	4.44	264.1362	264.1367	263.1289	245.1183	299.1056	309.1344	323.1500	153.0914	219.1382	204.1150	201.1269	203.1059	71
26	Ellagic Acid	$C_{14}H_6O_8$	4.87	302.0063	302.0068	300.9990	282.9884	336.9757	347.0045	361.0201	283.9935	300.9970	257.0060	229.0137	201.0191	71

ID	Compound name	Molecular Formula	Predicted tR (min)	Monoisotopic Mass	[M]⁻	[M-H]⁻	[M-H$_2$O-H]⁻	[M+Cl]⁻	[M+HCOOH-H]⁻	[M+CH$_3$COOH-H]⁻	Qual. 1	Qual. 2	Qual. 3	Qual. 4	Qual. 5	Reference
27	DL-p-Hydroxyphenyllactic Acid	$C_9H_{10}O_4$	4.25	182.0579	182.0585	181.0506	163.0401	217.0273	227.0561	241.0718	163.0392	135.0440	119.0490	134.0370	181.0499	72
28	Phenylacetic Acid	$C_8H_8O_2$	4.28	136.0524	136.0530	135.0452	117.0346	171.0218	181.0506	195.0663	135.0441	134.0362	107.0489	106.0411	136.0476	72
29	2-Methoxybenzoic Acid	$C_8H_8O_3$	3.05	152.0473	152.0479	151.0401	133.0295	187.0167	197.0455	211.0612						37
30	Trimethoxybenzoic Acid	$C_{10}H_{12}O_5$	3.41	212.0685	212.0690	211.0612	193.0506	247.0379	257.0667	271.0823						37
31	4-Methoxyphenylacetic Acid	$C_9H_{10}O3$	4.48	166.0630	166.0635	165.0557	147.0452	201.0324	211.0612	225.0768						37
32	Hesperetin	$C_{16}H_{14}O_6$	7.75	302.0790	302.0796	301.0718	283.0612	337.0484	347.0772	361.0929	301.0715	286.0463	164.0115	151.0029	242.059	73
33	Quercetin 3,3'-Dimethyl Ether	$C_{17}H_{14}O_7$	7.93	330.0740	330.0745	329.0667	311.0561	365.0434	375.0722	389.0878						74
34	Rosmarinic Acid	$C_{18}H_{16}O_8$	5.49	360.0845	360.0851	359.0772	341.0667	395.0539	405.0827	419.0984	197.0454	161.0240	359.0767	133.0291	135.0448	75
35	3-Hydroxybenzoic Acid	$C_7H_6O_3$	3.34	138.0317	138.0322	137.0244	119.0139	173.0011	183.0299	197.0455	93.0332	137.0233	94.0365	138.0268	56.3217	72
36	Dicaffeoylquinic Acid	$C_{25}H_{24}O_{12}$	7.19	516.1268	516.1273	515.1195	497.1089	551.0962	561.1250	575.1406						39
37	Prenyl Caffeate	$C_{14}H_{16}O_4$	9.12	248.1049	248.1054	247.0976	229.0870	283.0743	293.1031	307.1187						39
38	Rhamnetin	$C_{16}H_{12}O_7$	7.89	316.0583	316.0589	315.0510	297.0405	351.0277	361.0565	375.0722	315.0505	165.0194	121.0294	300.0279	193.0141	39
39	Kaempferide	$C_{16}H_{12}O_6$	7.88	300.0634	300.0639	299.0561	281.0455	335.0328	345.0616	359.0772	284.0323	299.0555	151.0037	164.0114	300.0624	39
40	Sakuranetin	$C_{16}H_{14}O_5$	8.06	286.0841	286.0847	285.0768	267.0663	321.0535	331.0823	345.0980	285.0779	165.0199	119.0506	286.0808	243.0664	39
41	Alpinetin	$C_{16}H_{14}O_4$	7.73	270.0892	270.0898	269.0819	251.0714	305.0586	315.0874	329.1031						39
42	Pinostrobin	$C_{16}H_{14}O_4$	8.23	270.0892	270.0898	269.0819	251.0714	305.0586	315.0874	329.1031						39
43	Tectochrysin	$C_{16}H_{12}O_4$	8.22	268.0736	268.0741	267.0663	249.0557	303.043	313.0718	327.0874	252.0408	267.0659	268.0684	224.0472	269.0648	39
44	Dihydroxyflavone	$C_{15}H_{10}O_4$	8.12	254.0579	254.0585	253.0506	235.0401	289.0273	299.0561	313.0718						39
45	DL-β-Phenyllactic acid	$C_9H_{10}O_3$	3.65	166.0630	166.0635	165.0557	147.0452	201.0324	211.0612	225.0768						72
46	Kojic Acid	$C_6H_6O_4$	3.01	142.0266	142.0272	141.0193	123.0088	176.9960	187.0248	201.0405	141.0187	111.0091	113.0238			76
47	Dehydrovomifoliol	$C_{13}H_{18}O_3$	5.42	222.1256	222.1261	221.1183	203.1078	257.0950	267.1238	281.1394						76
48	Benzoic Acid	$C_7H_6O_2$	2.94	122.0368	122.0373	121.0295	103.0189	157.0062	167.0350	181.0506	121.0304	92.0263	120.0213	93.0348		76
49	Lumichrome	$C_{12}H_{10}N_4O_2$	5.85	242.0804	242.0809	241.0731	223.0625	277.0498	287.0786	301.0942						76
50	Rutin	$C_{27}H_{30}O_{16}$	6.29	610.1534	610.1539	609.1461	591.1355	645.1228	655.1516	669.1672	609.1474	301.0366	300.0319	271.0274	343.0538	77
51	Isoquercetin	$C_{21}H_{20}O_{12}$	5.91	464.0955	464.0960	463.0882	445.0776	499.0649	509.0937	523.1093	463.0891	301.0363	300.0294	271.0248	151.0037	77
52	Sinapic Acid	$C_{11}H_{12}O5$	3.33	224.0685	224.0690	223.0612	205.0506	259.0379	269.0667	283.0823	149.0258	193.0166	208.0402	164.0498	223.0607	77
53	p-Methoxybenzoic Acid	$C_8H_8O_3$	2.89	152.0473	152.0479	151.0401	133.0295	187.0167	197.0455	211.0612	92.0263	136.0121	92.0155	141.7980		77
54	Morin	$C_{15}H_{10}O_7$	7.19	302.0427	302.0432	301.0354	283.0248	337.0121	347.0409	361.0565	151.0021	271.0255	301.0363	299.0208		77
55	p-Methoxycinnamic Acid	$C_{10}H_{10}O_3$	3.08	178.0630	178.0635	177.0557	159.0452	213.0324	223.0612	237.0768	177.0552	103.0551	133.0663	131.0510	105.0702	77

ID	Compound name	Molecular Formula	Predicted tR (min)	Monoisotopic Mass	$[M]^-$	$[M-H]^-$	$[M-H_2O-H]^-$	$[M+Cl]^-$	$[M+HCOOH-H]^-$	$[M+CH_3COOH-H]^-$	Qual. 1	Qual. 2	Qual. 3	Qual. 4	Qual. 5	Reference
56	4-Hydroxyacetophenone	$C_8H_8O_2$	4.36	136.0524	136.0530	135.0452	117.0346	171.0218	181.0506	195.0663						78
57	3,4-Dimethoxycinnamic Acid	$C_{11}H_{12}O_4$	3.32	208.0736	208.0741	207.0663	189.0557	243.0430	253.0718	267.0874	207.0657	103.055	163.0763	133.0654		78
58	Fisetin	$C_{15}H_{10}O_6$	6.68	286.0477	286.0483	285.0405	267.0299	321.0171	331.0459	345.0616	135.0093	285.0405	121.0287	255.0270	163.0029	79
59	Naringin	$C_{27}H_{32}O_{14}$	6.79	580.1792	580.1798	579.1719	561.1614	615.1486	625.1774	639.1931	271.0641	579.1728	151.0029	459.1191	459.1089	79
60	Hesperidin	$C_{28}H_{34}O_{15}$	6.97	610.1898	610.1903	609.1825	591.1719	645.1592	655.1880	669.2036	301.0736	609.1862	242.0585	151.0037	286.0483	79

Section S8: Level of identification confidence

The level of identification confidence for each detected compound was established, according to Schymanski et al [81]. According to this study, identification level 5 refers to the exact mass (*m/z*) of interest corresponding to an unknown compound. Level 4 is used for identification when a molecular formula can be assigned unambiguously to this *m/z*. If there is sufficient MS (mass accuracy, existence of isotopic peak or adducts pattern), experimental information (eg. t_R) and interpretable MS/MS fragments (by *in silico* fragmentation tool), each plausible candidate can gain level 3 in the identification confidence (tentatively identified compound). This level indicates that there is one or more possible structure(s) available for given information, but they are insufficient to eliminate other possible structural candidates (isomers etc.). If the experimental MS/MS match between the compound and the reference spectra in the spectral libraries (MassBank, GNPS, mzCloud or literature) or diagnostic evidence is present to discard other plausible candidates, the identification of this compound can reach level 2 (which means the most probable structure). Level 2 includes two sublevels; level 2a, which is when the MS/MS information of the detected compound matches with literature or spectral library and level 2b refers to diagnostic evidence, such as agreement between predicted and experimental t_R, distinct mass loss (because of small alteration of moieties in the chemical structure i.e. demethylation, N-oxides formation etc.) between parent and transformation compounds or existence of diagnostic MS/MS fragments. Finally, if all the MS, MS/MS fragments and t_R information match between the reference standard and the detected compound, the identification level is set to 1.
